# IMVEST, an immersive multimodal virtual environment stress test for humans that adjusts challenge to individual's performance

**DOI:** 10.1016/j.ynstr.2021.100382

**Published:** 2021-08-13

**Authors:** João Rodrigues, Erik Studer, Stephan Streuber, Carmen Sandi

**Affiliations:** a Laboratory of Behavioral Genetics, Brain Mind Institute, École Polytechnique Fédérale de Lausanne, Lausanne, Switzerland; b Virtual Reality for Collective Behaviour Group, Department of Computer and Information Science, University of Konstanz, Konstanz, Germany

**Keywords:** Stress test, Sympathetic nervous system, Virtual reality, Machine learning, Multimodal stress

## Abstract

Laboratory stressors are essential tools to study the human stress response. However, despite considerable progress in the development of stress induction procedures in recent years, the field is still missing standardization and the methods employed frequently require considerable personnel resources. Virtual reality (VR) offers flexible solutions to these problems, but available VR stress-induction tests still contain important sources of variation that challenge data interpretation. One of the major drawbacks is that tasks based on motivated performance do not adapt to individual abilities. Here, we provide open access to, and present, a novel and standardized immersive multimodal virtual environment stress test (IMVEST) in which participants are simultaneously exposed to mental -arithmetic calculations- and environmental challenges, along with intense visual and auditory stimulation. It contains critical elements of stress elicitation – perceived threat to physical self, social-evaluative threat and negative feedback, uncontrollability and unpredictability – and adjusts mathematical challenge to individual's ongoing performance. It is accompanied by a control VR scenario offering a comparable but not stressful situation. We validate and characterize the stress response to IMVEST in one-hundred-and-eighteen participants. Both cortisol and a wide range of autonomic nervous system (ANS) markers – extracted from the electrocardiogram, electrodermal activity and respiration – are significantly affected. We also show that ANS features can be used to train a stress prediction machine learning model that strongly discriminates between stress and control conditions, and indicates which aspects of IMVEST affect specific ANS components.

## Highlights

• Laboratory stressors are an essential tool to study the stress response in humans.
• We present a novel immersive multimodal virtual environment stress test (IMVEST).
• IMVEST adapts to individual performance.
• Induces acute increase in stress markers.
• Stress responses do not depend on performance differences.

## Graphical abstract

**Figure:**
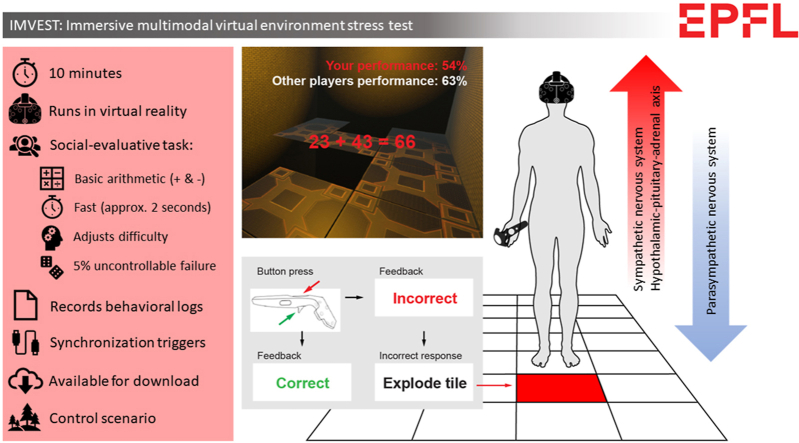

## 1 Introduction

Stress can have a profound impact in a myriad of physiological systems. Although a lot of research has been devoted to chronic stress given its deleterious impact on physical and mental health (Juster et al., 2010; Lupien et al., 2018; Sandi, 2004; Tamashiro et al., 2011), the understanding of acute stress – in terms of both effects and mechanisms – in humans is lagging behind. Preclinical work suggests that exposing subjects to acute stress can be an effective probe to deliver biomarkers capable of predicting vulnerability to develop psychopathologies (Cordero et al., 1998; Daskalakis et al., 2016; Hodes et al., 2014; Nasca et al., 2019; Walker and Sandi, 2018). In addition, acute stress responses can on their own affect brain function, cognition and behavior (De Quervain et al., 2016; Sandi, 2013; Schwabe, 2017) and affect numerous body systems (Floriou-Servou et al., 2021; Pfau and Russo, 2015). Therefore, progress on understanding how humans respond to stressful challenges acutely is crucial for the advancement of individual and population health (Allen et al., 2017). However, at difference to chronic stress that – for ethical reasons, given its damaging effects – rely on life experiences, studies on acute stress are typically performed in the laboratory and, therefore, depend on the development of effective procedures capable of generating sufficient level of stress without causing undesirable long-term effects on participants.

The physiological acute stress responses aim at facilitating adaption to threats, and involve the activation of the sympathetic branch [i.e., sympathetic nervous system (SNS)] of the autonomic nervous system (ANS) and the hypothalamic-pituitary-adrenal (HPA) axis (Joëls and Baram, 2009; Ulrich-Lai and Herman, 2009). SNS responses are promptly triggered following exposure to stressors leading to immediate increases in heart, electrodermal activity and respiration rates (Barry and Fureby, 1993; Joëls and Baram, 2009; Ulrich-Lai and Herman, 2009), while reduced vagal activity from the parasympathetic branch of the ANS leading to lower heart rate variability (HRV) (Ottaviani, 2018). Conversely, increased blood levels of glucocorticoids (cortisol, in humans), the final products of the activated HPA axis, become apparent only around 10–15 min after stress onset (De Kloet et al., 2005).

Several laboratory stress-induction methods [e.g., the cold pressor test (CPT; (Hines, 1937)); the socially evaluated cold-pressor test [SECPT; (Schwabe et al., 2008)], the Maastricht acute stress test [MAST; (Smeets et al., 2012)], and the Trier social stress test [TSST; (Kirschbaum et al., 1993)] have been developed to study the acute stress response in humans and yielded invaluable knowledge on the impact of acute stress on many health-related domains [for reviews, see (Allen et al., 2014; Helminen et al., 2019; Lai et al., 2021; Miller and Kirschbaum, 2019; Mücke et al., 2018; Paris et al., 2010; Schwabe and Schächinger, 2018; Seddon et al., 2020; Shields et al., 2017; Slavish et al., 2015; Steptoe et al., 2007; Wolf, 2019)]. These methods differ in their respective efficiency and reliability in triggering physiological stress responses (Dickerson and Kemeny, 2004), providing to the field a rich pipeline of diverse stressor protocols beneficial for the external validity of stress research. However, improving consistency of methodology across laboratories can also be instrumental for comparability of data and to allow the performance of meta-analyses.

In the laboratory, psychological stressors (e.g., TSST, SECPT, MAST) have proved to be more effective to elicit robust stress reactions than physical stressors (e.g., the CPT). Specifically, elements of uncontrollability and social-evaluative threat (i.e., when task performance is under evaluation by other persons) are particularly effective (Dickerson and Kemeny, 2004). Among current methods, the TSST – consisting of a mock job interview followed by arithmetic mental calculations while the participant is evaluated by 2–3 committee members trained to act neutrally – (Kirschbaum et al., 1993) is considered the gold standard of assessment of acute stress under laboratory conditions (Allen et al., 2017). Although the TSST represents a robust and standardized paradigm to trigger psychophysiological stress, it contains important sources of variation [(e.g., number of judges, characteristics of the arithmetic task; see also below) that challenge some aspects of data meta-analyses interpretation (Allen et al., 2017; Narvaez Linares et al., 2020)]. To account for these issues, several virtual reality (VR) versions of the TSST (TSST-VR) have been developed with the goal of increasing standardization and use of fewer resources (e.g., dedicated personnel as committee members) (Helminen et al., 2019).

With recent technological developments and task refinement, TSST-VR tasks are progressively becoming effective to trigger stress responses [i.e., cortisol, heart rate (HR), and self-report] comparable to the traditional TSST (Helminen et al, 2019, 2021; Zimmer et al., 2019). However, these VR tests do not account for differences in individuals' capacity in public speaking or mathematical skills, and therefore, it is not possible to disentangle variation in stress responses due to differences in stress reactivity vs differences in individual's capabilities in the specific task demands. Accounting or adjusting for the public speaking/interview part is not possible due to the broad range of human experiences that can be relevant for interview settings, and the fact that participant's linguistic complexity is linked to TSST stress reactivity (Saslow et al., 2014).

In contrast, mathematical challenges are amenable to be manipulated to individual's performance levels. Precisely, manipulation of an arithmetic task to be slightly above the participant's level of mathematical skill principle has been implemented in the Montreal imaging stress task [MIST; (Dedovic et al., 2005)], a version derived from the TSST (without a job interview component) that combines computerized mental arithmetic challenges along with social evaluative threat components, and elicits SNS and cortisol responses (Jones et al., 2011; Voellmin et al., 2015). However, MIST was built to be delivered in scanners during neuroimaging studies and is, therefore, presented through 2D screens while participants keep a static posture. It is known that, compared to immersive tasks delivered in VR, 2D screen-delivered tasks trigger lower levels of ‘presence’ (Kober et al., 2012; Roettl and Terlutter, 2018). Presence is an important sensation to feel stress in laboratory tasks (Morina et al., 2014), and is facilitated in immersive VR environments as it offers a continuous spatial and temporal experience that facilitates the participant's subjective feeling of presence (Metzinger, 2018) and the sensation of ‘being there’ (Slater, 2003). In addition, performance in 2D screens while participants are seating or laying down prevents from obtaining information of participant's body movements and spatial navigation, and these features are emerging as highly informative of individual's vulnerability to stress (Rodrigues et al., 2020).

Here, we describe a novel immersive multimodal virtual environment stress test (IMVEST), delivered in VR, that adjusts the challenge to performance of the individual and contains important elements of stress elicitation, such as social-evaluative threat and uncontrollability. In addition, it is multimodal stress because participants are simultaneously exposed to mental and environmental challenges, along with intense visual and auditory stimulation. Specifically, participants are demanded to rapidly respond to arithmetic calculations where failure to do so accurately results in a penalized response with negative performance feedback and delivery of aversive stimuli. Furthermore, in order to avoid task habituation, we introduced an additional negative feedback component at the middle of the task where participants are informed that, due to their poor performance, the test has failed and the task needs to be restarted. The scripted nature of IMVEST provides flexibility for laboratories with different languages to use it while the automatic adjustment of difficulty provides consistency in the stressor applied across laboratories. Importantly, the software records a log of the participant's positioning and choices along with detailed information of each trial that can be used for behavioral analyses. It can also be configured to send triggers to external sources for synchronization with telemetry equipment or other devices. Finally, the stress test is accompanied by a control VR scenario offering a comparable but not stressful situation.

To validate IMVEST and characterizing the elicited stress responses [i.e., salivary cortisol, SNS – electrocardiogram (ECG), electrodermal activity (EDA) and respiration – and behavioral responses], we tested 118 participants that were exposed to either the stressful or the control task. We show that IMVEST is an effective stress procedure triggering increases in cortisol and SNS changes, and demonstrate that the implemented difficulty adjustments ensure its effectiveness regardless of individuals’ mathematical competence. Furthermore, by applying machine learning methods to ANS variables, we show a high discrimination between stress and control conditions and illustrate how this approach can deliver an individualized stress score.

## 2 Materials and methods

### 2.1 The IMVEST

IMVEST is a Windows® application developed in the Unity® game engine and coded in the C# programming language, designed to run with an HMD with controller (HTC Vive®, Seattle, USA). It can be downloaded from (https://doi.org/10.5281/zenodo.4923695).

**Fig. 1:**
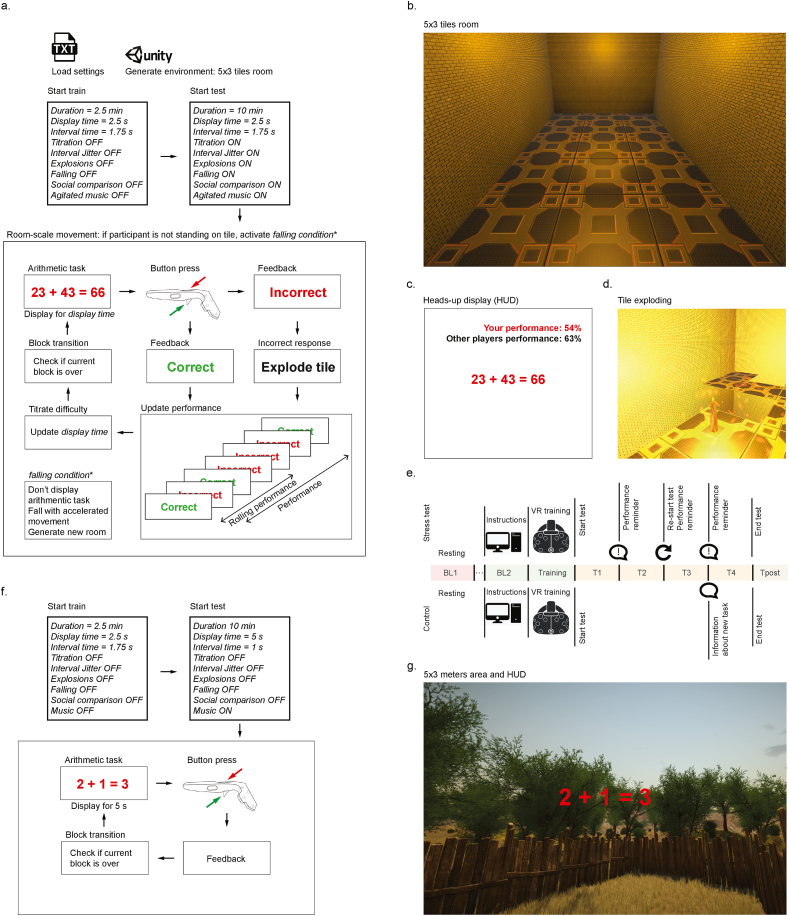
Description of IMVEST main elements. (**a**) Schematic for the logic governing IMVEST (consult Appendix 1-Fig. S1 for a more detailed flowchart). Settings are loaded from a text file and a room of 5 by 3 m is generated with the Unity® game engine. A 2.5 min training period precedes the 10 min test. During training, stressful elements are turned OFF, difficulty is not adjusted and intervals between arithmetic tasks last exactly 1.75 s without jitter. For the test, all these elements are turned ON. The test contains arithmetic tasks that are presented on screen during a certain amount of time (*display time*). During this time, the participant can respond pressing the controller trigger if the equality is true, or the controller button if the equality is false. Failing to respond within the *display time* is taken as an incorrect response. Incorrect responses trigger the explosion of a random tile in the room, leaving an open gap on the virtual floor. After each arithmetic task, the two performance variables, an overall measure of performance and a measure of rolling performance of the las 5 trials, are updated. The latter is titrated to be bound between 40% and 60% by incrementing or decrementing the *display time* variable, making the task slightly easier or difficult, respectively, depending on individual's performance. (**b**) Screenshot of the 5 by 3 tiles virtual room. (**c**) Example of the heads-up display (HUD) which is always present in the participants' field of view. Performance is presented in red if below 63%, the faux average of other participants, or green otherwise. (**d**) Example of a tile exploding. (**e**) Structure of the required experimental blocks to run the IMVEST or the control scenario. Blocks BL1 and Tpost allow acquiring information about baseline under resting conditions and a post-stress short recovery period; both blocks can be expanded if required. Instructions during BL2 are presented in the computer screen. The remaining blocks are in VR. After blocks T1, T2 and T3, the stress test is paused and a pre-recorded voice informs participants that they need to perform at their best for the experimental results to be valid. In addition, at the end of block T2, participants are informed that their performance was not good enough and consequentially the test would have to restart. For the control scenario, the arithmetic task stops after T3 and participants are allowed to freely manipulate environmental aspects of the simulation (i.e., play with differential accelerations of the day-night cycle) to avoid task habituation. (**f**) Schematic for the logic governing the control VR scenario offering a comparable but not stressful situation, without stressful elements and with a positive valence brought by the nature depiction and a low arousal and pleasant music. Arithmetic tasks are easier and presented for longer than in the stress scenario. (**g**) Screenshot of the nature setting used for the control scenario. (For interpretation of the references to colour in this figure legend, the reader is referred to the Web version of this article.)

IMVEST makes use of the safety and controllability of a virtual environment to threaten central goals of physical and social self-preservation with a cognitive and navigational laboratory challenge. We designed IMVEST to induce acute stress in humans following recommendations to elicit physiological responses when applying acute psychological stressors in the laboratory (Dickerson and Kemeny, 2004), IMVEST comprises a motivated performance task aimed at: 1) creating a context of forced failure by performance titration with consequences for negative performance; 2) socio-evaluative pressure; 3) unpredictability; 4) uncontrollability; and 5) a cognitive component. In addition, and following evidence that multimodal stressors are more effective than unimodal ones (Maras et al., 2014), IMVEST contains sensory stressors, of both audio (explosions, music) and visual modalities. See Fig. 1a–d for more details.

#### 2.1.1 Virtual environment

The test is performed at room-scale in a virtual room of 15 m^2^ with 3 × 5 square tiles (1 m^2^ each). Each tile can explode, leaving an open gap on the floor through which the participant can fall (falling velocity is accelerated by a factor of 1.2 each 16 ms) and land on a new room, with the same dimensions as the previous. When an explosion event is triggered, a 1 s alarm rings while the selected tile blinks twice red, followed by a 0.75 s explosion animation that makes the tile fall. Falling, recreated by gravitational accelerated movement, brings participants to a new room where the arithmetic challenge resumes. Given the number of trials in the task, falling inevitably happens either due to a navigation mistake or lack of tiles to stand on.

There is no limit to the number of rooms that can be generated shall the participant fall more than once; the test only ends after the time limit is reached. While immersed in VR, participants can see where they stand in the virtual room and walk around within its limits and avoid falling into the eventual open gaps. If all tiles disappear, falling in the ‘virtual space’ is inevitable.

The participant's body position in the virtual scenario is approximated from the HMD location (which is also the camera coordinates) by being 10 cm behind it. This position is used to determine if the participant is on top of a tile or on top of a gap, and hence should fall. Simultaneously, text is presented in the HUD located in the central field of view. The HUD shows the participant's current overall performance, a fake average performance of other participants to create constant socio-evaluative pressure, the mathematical formulas and feedback about each response. During the training block, informative text is also presented in the HUD. When the performance lies below the fake average of other participants, which was set to 63% for this study, the text in the HUD displaying their performance turns red, otherwise it is green.

#### 2.1.2 Structure of the arithmetic trial

A stressful cognitive component is elicited with the simultaneous task demand of navigation and solving mental arithmetic tasks known to effectively induce stress (Dedovic et al., 2005; Pruessner et al., 1999). In each trial, a mathematical formula is presented on the HUD, which the participant has to identify if correct or incorrect by pressing one of the controller's buttons. Additions and subtractions were used to allow for fast responses. The formula is displayed on the HUD for 2.5 s during which, a button should be pressed. Failure to press the button (timeout) is considered an incorrect response. Besides decreasing the participant's performance, incorrect responses trigger a random tile to explode, contributing to the unpredictable component of this challenge and augmenting the threat to the physical self-preservation.

Participants’ performance is continuously adjusted as a function of their ongoing performance as follows: Depending on performance, the display time in the visual field in VR, that starts at 2.5 s, is adjusted throughout the experiment to keep the running performance (measured as the rate of right responses in the previous five trials) between 40% and 60%. This is done with either increments or decrements of 60 ms in the following way:

- If running performance is >60% and display time >1 s: subtract 60 ms from display time (i.e., leading to ‘decrements’ in time).
- If running performance is <40% and display time <3 s: add 60 ms to display time (i.e., leading to ‘increments’ in time).

Inter-trial intervals last on average 1.75 s and are jittered to prevent trial anticipation by randomly varying this length by – 0.525 s–0.525 s. To further increase the sense of uncontrollability and unpredictability, there is a 5% chance that a correct response is considered as incorrect.

#### 2.1.3 Block structure

The test was programmed in four 2.5-min blocks, each comprised of several arithmetic trials (see Fig. 1e and flowchart for the program logic in Appendix 1-Fig. S1). At the end of blocks T1, T3 and T4, a pre-recorded voice asked participants to improve their performance so that their data can be used. In addition, at the end of T2, which separates the two halves of the test, they were informed that given their insufficient performance, the test was not valid and needed to be restarted. At this point, they were also demanded to improve their performance. Restarting resets all the variables such as performance and display time to their initial values and restores all floor tiles. Thus, T3 started with all elements reset to the same level as at the onset of T1.

#### 2.1.4 Audio elements

Sound elements include the alarm and explosion sounds that accompany a tile explosion, voice feedback and music. Throughout the test, the “Sonata for 2 pianos and percussion (Assai lento)” by the composer Béla Bartók is playing as it is classified as high arousal, negative valence (agitated) (Russo et al., 2013).

#### 2.1.5 Preparation for the test

In the first 2.5 min preparation period (BL2) participants sat in front of a computer screen and were instructed about the upcoming task via presentation slides. This presentation provided the necessary description of the task and controller button assignment but also informed participants their performance would be recorded, evaluated and compared to that of the other participants, hence ensuring socio-evaluative pressure. In the next 2.5 min (training) participants were equipped with the HMD and controller and the scenario was launched, starting with four training trials, no exploding tiles nor falls and the display time is always kept at 2.5 s, to get acquainted to the controller and button assignment. After, participants were presented with an example of a tile exploding and disappearing, and were instructed by text on the HUD not to fall in the resulting gap. The remaining time was spent with more training trials.

### 2.2 The control scenario

This scenario consists of a VR immersion with similar elements to the stress test but without any of the stressful elements: 1) lack of forced failure or consequence to incorrect responses; 2) lack of socio-evaluative pressure; 3) predictability; 4) controllability; 5) a low cognitive component. See Fig. 1f–g for details.

#### 2.2.1 Virtual environment

The control task consists on the immersion in a virtual nature setting surrounded by trees and, to avoid habituation and boredom, it contains a day-night cycle. Participants are free to navigate within the limits of a room-scale yard consisting of a 15 m^2^ (3 m × 5 m) delimited by a virtual wooden fence, placed to prevent collision with the laboratory walls. Simultaneously, HUD shows only support text, the mathematical formulas and feedback about each response. During the last 2.5 min block (T4), participants were allowed to control environmental aspects of the virtual world to avoid task habituation. Performance is not recorded nor displayed.

#### 2.2.2 Structure of the arithmetic trial

As in the stress test, additions and subtractions were used but with only one-digit numbers. The display time on the HUD is kept at a longer period of 5 s and not adjusted. The inter-trial interval is also fixed, at 1 s. Response feedback is still provided and a lack of button press (timeout) is considered an incorrect response. While it is still possible to fail and receive negative feedback, there is no associated negative consequence.

#### 2.2.3 Block structure

The scenario was also programmed in four 2.5 min blocks (see Fig. 1e). The first three blocks were identical and consisted of calculation trials. By the end of the third calculation trial block, positive feedback was given by congratulating participants for their previous performance, and informing that they could now control the day-night cycle in the virtual environment. Hence, the fourth block gives controllability to participants by allowing them to accelerate the passage of time in the day-night cycle by keeping a button pressed.

#### 2.2.4 Audio elements

Sound elements include nature sounds and throughout the immersion, the “Flute and Harp Concerto in C, K. 299, 2nd Movement” by W.A. Mozart is played as it is classified as high positive valence and low arousal (Lynar et al., 2017).

#### 2.2.5 Preparation for the test

In the first 2.5 min preparation period (BL2) participants sat in front of a computer screen and were instructed about the upcoming task via presentation slides. This presentation provided the necessary description of the task and controller button assignment. No performance recording is mentioned. In the next 2.5 min (training) participants were equipped with the HMD and controller, the scenario was launched and they were allowed to perform practice trials until the end of this block.

### 2.3 Software input and output

The IMVEST application runs by default with the settings described in Fig. 1a. However, some of these variables can be specified in a settings file: Initial display time, interval time, increment/decrement of display time and titrated performance boundaries. The audio elements can also be changed. The software also requires a text file with the participant number, which if left empty a random identifier is used instead. The IMVEST application can send triggers (10 ms pulse with numbered events) through a serial-port for synchronization with external devices. The port number needs can be specified in the settings file. Log files are also generated at each run with information about the system time (in the computer running the software), cartesian coordinates of the HMD and controller, button presses, trial number, trial info (right or wrong formula), arithmetic formula in string format and current tile participant is standing on.

### 2.4 Experimental setup

Virtual reality was performed using an HMD (HTC Vive®, Seattle, USA) and controller (HTC Vive controller®, Seattle, USA). The dimensions of the testing room are 3.50 m (width), 6.00 m (length), and 3.50 m (height). Within this range, participants could move around freely. Virtual room dimensions (walls) were kept smaller than the room's physical walls to avoid collisions. We ensured that participants could never see the physical room to increase immersion and the sense of novelty when immersed in the virtual scenarios.

We used a wireless physiology system (Biopac Bionomadix) recording data at a 1000 Hz sampling rate with AcqKnowledge Data Acquisition and Analysis Software 5.0. We recorded respiration, ECG and EDA. ECG was kept at 1000 Hz while respiration and EDA signals were decimated to 100 Hz using the MATLAB function *decimate*, with the default FIR filtering prior to down-sampling.

### 2.5 Experimental validation of the IMVEST stress elicitation

#### 2.5.1 Participants

One hundred eighteen (118) male participants between the ages of 18 and 38 (age: 20.5 ± 2.06 years) were recruited and randomly assigned into Experimental/Stress (N = 57 subjects, age: 20.4 ± 2.14 years) and Control/No-stress (N = 61 subjects, age: 20.7 ± 2.05 years) groups. Criteria for inclusion in the study included being healthy, male, 18–38-year-old, French-speaking, non-smoking, not under psychotropic or hormonal medication, no history of neurological, psychological or cardiac disease and no corrected vision (with glasses). For this first validation study of IMVEST, we focused in men aiming at a having a rather homogeneous cohort (i.e., devoid of potential variation associated with the female menstrual cycle) that would allow us using an affordable sample size to assess the effectiveness of the key test manipulations (i.e., the performance adjustment -see section 2.1.2- and the negative feedback provided right after completion of block T2 -see section 2.1.3). Future work will address a comparison between male and female responses to IMVEST.

Exclusion criteria consisted of self-reported psychiatric, neurological and medical conditions, having consumed alcohol or illegal drugs recently. After receiving a complete description of the study, each participant gave informed written consent to participate. Participants were given a financial compensation of CHF 20 per hour. The study was approved by the Cantonal Ethics Committee of Vaud, Switzerland (CER-VD). Days before the experiment, participants were asked to complete a demography questionnaire (age and fluency in the questionnaires and experimental language – French), the Spielberger Trait Anxiety Inventory [STAI-T, form Y (Spielberger, 1983)], the Social Interaction Anxiety Scale [SIAS, (Mattick and Clarke, 1998)] and to perform a custom-made cognitive ability test consisting of a 10-min timed version of the Bochumer Matrizen-test (Goette et al., 2015). For the purpose of salivary cortisol measurement, participants were asked not to eat or drink beverages other than water 1 h prior to the experiment. Participants were also asked to refrain from strenuous exercise and alcohol in the 24 h before the experiment. All experiments occurred after 1 p.m. to avoid the morning circadian influence on cortisol and before 7pm.

#### 2.5.2 Experimental procedure

The experiment started after participant arrival, once they gave informed consent. A saliva sample was taken (s1) to assess cortisol levels upon arrival and participants were equipped with wireless sensors to measure ECG, EDA and respiration. After equipment calibration and signal quality check, signal baselines were recorded with participants seated on a chair in front of a computer screen and remaining still for 2.5 min. Baseline levels of state-anxiety and positive and negative affect were then obtained by collecting responses to the Spielberger State Anxiety Inventory [STAI-S, form Y (Spielberger, 1983)] and the Positive and Negative Affect Schedule [PANAS, grouped into a positive and negative variables; (Crawford and Henry, 2004) questionnaires respectively. Still in front of the computer screen, participants had 2.5 min to read the instructions for the experimental task (stress or control; block BL2), after which they were equipped with the HDM and controller and taken to the center of the experimental room. Prior to the start of the test, a 2.5 min training (block training) was given to explain the mechanics of the test environment and allow participants to get acclimated to VR and familiar with the controller and button assignment. The test lasted 10 min and immediately after its end, a saliva sample was collected (s2). During the 7.5 min preceding saliva sample s3, participants filled a self-report of the Igroup Presence Questionnaire [IPQ; (Schubert et al., 2001)], from which four presence variables were extracted (general presence, spatial presence, involvement and experienced realism). After collection of saliva sample s3, equipment was removed and participants waited a recovery period to take saliva sample s4.

### 2.6 Physiology analysis

In order to facilitate reproducibility and obtain a large number of ANS features, electrocardiography variables were computed with two open source MATLAB toolboxes [HRVTool (Vollmer, 2019) and PhysioNet Cardiovascular Signal Toolbox (Vest et al., 2018)]. More specifically, normal-normal (NN) intervals are identified with the PhysioNet Cardiovascular Signal Toolbox (see Appendix 1-Table 1 for details on detection and quality thresholds) and used by both toolboxes to compute the electrocardiography variables. Skin conductance variables were also computed with an open source MATLAB toolbox, Ledalab (Benedek and Kaernbach, 2010). Respiration variables were computed using the MATLAB function *findpeaks*. We identified peaks above at least one standard deviation (robustified) of the filtered respiration time series, separated by at least 0.8 s and with a minimum width of 0.4 s. Besides median respiration rate, we also median extracted peak prominence and width to compute a variable with their ratio (prominence/width).

A total of 25 physiological variables were used. Details and explanations can be found in Table 2. Variables were computed for each 2.5 min block (BL1, BL2, training, T1-4, Tpost).

**Table 1:** **Demographics and personal traits.** French fluency – an inclusion/exclusion criterion of the study – was assessed in a scale from 0 (non-existent) to 10 (native). Differences between Control and Stress groups assessed with Mann-Whitney U tests.

	Group	N	Mean	Standard deviation	Mann-Whitney U	p-value
Age	Control	61	20.7	2.1	1578	0.382
	Stress	57	20.4	2.1		
French Fluency	Control	61	9.6	1.1	1716	0.853
	Stress	57	9.7	0.8		
Trait-anxiety	Control	61	35.9	7.2	1732	0.974
	Stress	57	36.0	7.3		
Social anxiety	Control	61	25.6	13.9	1610	0.489
	Stress	57	24.0	14.2		
Cognitive ability	Control	61	0.25	0.07	1724	0.937
	Stress	57	0.25	0.07

**Table 2:** **Physiology variable information:** sensors used for measuring, measurement name, brief explanation, variable name and toolbox used to compute it.

Sensor	Measurement	Explanation		Variable name	Toolbox
Electrocardiogram (ECG)	Heart Rate (HR)	HR in beats per minute		HR	HRVTool
	Heart rate variability (HRV)	Poincaré plots	Short-term variability	SD1	PhysioNet
			Long-term variability	SD2	
			Ratio between SD1	SD1SD2	
		Geometric methods based on the normal-normal (NN) histogram	Baseline width of the RR interval histogram	TINN	HRVTool
			Area of RR interval histogram/height	TRI	
		Phase-rectified signal averaging	Acceleration capacity	ac	PhysioNet
			Deceleration capacity	dc	
		Entropy methods	Sample entropy	SampEn	
			Approximate entropy	ApEn	
		Frequency domain methods	Low frequency	lf	
			High frequency	hf	
			Ratio	lfhf	
			Total power	ttlpwr	
		Linear time domain	Root mean square of successive differences of the RR intervals	RMSSD	
			Standard deviation of the RR intervals	SDNN	
			Percentage of NN differences greater than 50 ms	pnn50	
			Normalized HRV	rrHRV	HRVTool
Breathing belt	Respiration	Respiration rate as cycles per second		RespRate	MATLAB's findpeaks
		Height of respiration cycles		RespDepth	
		Length of respiration cycles		RespWidth	
		Ratio between respiration width and depth		RespRatio	
Electrodermal activity (EDA) electrodes	Non-specific skin conductance (not associated to stimuli or events)	Number of skin conductance responses (SCR) in 1 min (using continuous decomposition analysis)		nSCRcda	Ledalab
		Number of SCRs in 1 min (using trough to peak analysis)		nSCRttp	
		Tonic level of EDA.		Toniccda	

A baseline (BL1) removed version of each variable (denoted with the prefix Δ) was also computed from each block (BL2, training, T1-4, Tpost) by subtracting the corresponding value at BL1.

### 2.7 Cortisol analysis

Cortisol was sampled 4 times: Arrival (s1), right after the end of the main test (s2), 20 min after the end of the main test (s3) and after a recovery period, 40 min after the end of the main test (s4). To account for cortisol changes through time, circadian influence in cortisol was corrected similarly to (Elbau et al., 2018) by subtracting, for each subject, the line between s1 and s4 from each cortisol sample. The AUC_i_ formula (Pruessner et al., 2003) was applied to the two corrected cortisol values after the stress task (s2 and s3) to estimate the total cortisol released between these two samples. Exact sampling times were obtained for the formula computation and averages were used for one participant where exact times were missing.

### 2.8 Behavioral and difficulty adjustment variables

Behavior was assessed via button presses and position tracking. Performance variables were measured from their responses by checking response accuracy, latency to respond and the ratio between these two as task competence. With position tracking, we computed variables regarding where participants positioned themselves in the virtual space [time in center area (3 center tiles), edges (12 peripheral tiles) and corners (4 corner tiles)], distance travelled, number of tiles visited and number of times they (virtually) fell. Difficulty adjustments done by the software to keep performance between 40% and 60% were also recorded as the percentage of times the formula display time was incremented (60 ms added) or decremented (60 ms subtracted).

### 2.9 Statistical analysis

rmANOVA was used and followed by post-hoc t-tests if the interaction term was significant. Sphericity assumption violations were tested with the Mauchly's test of sphericity and Greenhouse-Geisser corrections were applied whenever assumptions were violated. Pairwise comparisons were done with t-tests. One-sided t-tests were used whenever the direction of the effect was anticipated. Both normality and homogeneity of variances were assessed, respectively, with the Shapiro-Wilk and Levene's test. When these tests warned about violations of normality and homogeneity of variances, the Mann-Whitney *U* test or the Welch's *t*-test were also performed. For the correlation analysis, Spearman rank correlations were used throughout and rm-correlations were used whenever intra-subject associations were being studied (Bakdash and Marusich, 2017). Correction for multiple comparisons was applied with the Benjamini & Hochberg procedure for controlling the false discovery rate (Benjamini et al., 1995). Outlier detection and replacement were done in the following way: a value that was more than three scaled median absolute deviations away from the median of its variable was considered an outlier; outliers were replaced by the median of that variable (without the identified outliers). For more details please consult Appendix 1.

#### 2.9.1 Statistical software

Statistical analyses were performed using Jamovi v1.6.15.0, Python v3.7.1 (SciPy v1.6.1 and Pingouin v0.3.10). Outliers were identified and replaced using the MATLAB function *filloutliers* with default options.

### 2.10 Classification of IMVEST physiological stress responses

#### 2.10.1 Partial least squares regression discriminant analysis

Aiming at keeping our data analysis as simple and reproductible as possible, we chose a modeling approach that is data agnostic, requires minimal amount of hyper-parameter tuning and is based on the partial least squares (PLS) framework (Wold, 1985) and already widely used by the social sciences, bioinformatics, medical science and neuroscience [for more info (Rosipal and Krämer, 2006)]. PLS methods rely on the assumption that the response matrix Y being modeled is generated by a process that is driven by a small number of latent components, which are lower dimension projections of the original predictor variables X. Hence, in general PLS projects both X and Y into a lower-dimensional subspace such that the covariance between these latent components is maximal. This applies to when is only one response variable (PLS1) or two or more response variables (PLS2). PLS applied to regression problems (PLSR) can be seen as a multivariate high-dimensional regression method and is particularly suited for datasets with more predictor variables than observations and, relevant in our dataset, when there is multicollinearity among the predictor variables (Wold et al., 1984). PLSR allows for dimensionality reduction by restricting its prediction from an optimal reduced number of latent components determined by cross-validation.

PLSR was performed in Python using the *PLSRegression* function from the scikit-learn software package for a one-dimensional response variable (PLS1). Since PLS can be used in discrimination tasks (Barker and Rayens, 2003), we followed the procedure in its original implementation for discriminant analysis (Ståhle and Wold, 1987) where the one-dimensional response variable Y is a 0–1 dummy variable, where 0 denotes the control group and 1 denotes the stress group.

#### 2.10.2 Characterization of individuals by the number of physiological stress responses

Classifiers can be trained to distinguish between stress and control groups with physiological variables at each block. After training, a classifier should be able to classify new samples of variables as belonging to a stress block or to a control block. If such a classifier is expected to produce accurate predictions in new samples (based on cross-validation performance in during its training) subjects could then be characterized by the number of blocks (T1-T4) predicted as belonging to stress. Hence, to quantify each individual's physiology response we used a LOO procedure where, for each one of the *N* participants and *M* experimental blocks, a PLSR model was trained by fitting the physiological variables of the *M* experimental blocks from all the other *N*-1 participants to their corresponding experimental group (control or stress). This model was then used to predict each one of the four experimental blocks (T1-T4) from the current participant's physiology data to either belonging to a control or a stress group. To find an optimal threshold for the classifier's decision, each individual model was LOO cross-validated (CV) and its CV predictions were used to build receiver operating characteristic (ROC) curves. These ROC curves were then used to obtain an optimal decision threshold for each individual that maximizes the Youden's J statistic. The number of blocks classified as belonging to the stress group is the number of physiological stress responses which range from zero (all blocks T1-T4 were classified as belonging to the control group) to four (all blocks T1-T4 were classified as belonging to the stress group). See Appendix 1-Algorithm 1 for the pseudo-code description.

## 3 Results

### 3.1 IMVEST design and experimental setup

See the Methods section for complete details on the task. In addition, note that a key component of IMVEST that has not been implemented in any previous VR stress test is that, similarly to the 2D-screen task MIST (Dedovic et al., 2005), IMVEST titrates difficulty of the arithmetic task, to keep performance below the faux average. To increase the sense of uncontrollability, there was a 5% chance of a correct response to be taken as false. See Fig. 1a for a simplified depiction of the simulation.

The VR scenario (see Supplementary Video 1) consisted of a virtual closed room with tiled flooring (Fig. 1b) in which participants were prompted with fast mental arithmetic problems delivered through the heads-up display (HUD) (see Fig. 1c for an example). A wrong response resulted in the explosion of a floor tile (Fig. 1d), leaving a hole in the virtual floor through which participants could ‘virtually’ fall. The possibility of falling in VR elicits a threat to the physical self-preservation (Baker et al., 2020; Biedermann et al., 2017; Cleworth et al., 2012; Martens et al., 2019; Peterson et al., 2018; Seinfeld et al., 2016).

Supplementary video related to this article can be found at https://doi.org/10.1016/j.ynstr.2021.100382

The following is the supplementary data related to this article:

Video 1 Video 1

Social evaluative threat was evoked by visual information providing a permanent comparison of individual's performance in the arithmetic task with a faux average performance of ‘hypothetical’ previous participants (Fig. 1c) and by informing participants during the instructions that behavior would be recorded with video and their performance registered, evaluated and compared to other participants. The faux average performance of ‘hypothetical’ previous participants was set to 63% and feedback about participants' performance was presented in green if above this value and in red if below.

For analysis purposes, the experiment was divided into 8 blocks (BL1, BL2, Training, T1-T4, Tpost; see Fig. 1e). See section 2.1.3 for information on the different feedback manipulations.

In order to validate the stress-eliciting effectiveness of IMVEST, we developed a control scenario containing equivalent virtual immersion conditions as the stress scenario (Fig. 1e) without the stressful elements (see Fig. 1f, Supplementary Video 2 and Materials and Methods for details). In this task, participants perform simple mental arithmetic tasks with a long-time limit in a virtual nature setting (Fig. 1g) and there is no indication of participants’ performance.

Supplementary video related to this article can be found at https://doi.org/10.1016/j.ynstr.2021.100382

The following is/are the supplementary data related to this article:

Video 3 Video 3

Following exposure to the virtual environment test, participants were asked whether they had experienced motion sickness or dizziness. There were no reported or observable side-effects.

### 3.2 Physiological and behavioral responses to IMVEST exposure

#### 3.2.1 Assessment of cortisol, autonomic nervous system, and behavioral parameters

The primary endpoint of our study was to verify that IMVEST triggers changes in the standard physiological markers of stress; i.e., cortisol and key parameters of the ANS. A power analysis indicated that a sample size of at least 102 participants is required to detect a difference with an average effect size (Cohen's D of 0.5) between two groups with a power of 80%. To account for possible data exclusion, 118 participants were tested and split into an experimental/stress (N = 57) that performed the IMVEST and a control/no-stress (N = 61) group that performed the control scenario. The two groups were similar with regards to age, cognitive ability, trait-anxiety and social anxiety (all p > 0.382; see Table 1 for group demographics). Reported levels of state-anxiety, positive affect and negative affect right before test instructions were also similar between groups (all p > 0.167, Appendix 1-Table 2 for descriptive statistics). Reported levels of presence (general presence, spatial presence, involvement and experienced realism) were also similar between groups (all p > 0.270; see Appendix 1-Table 3 for descriptive statistics) and high (general presence in the stress group: 4.87 ± 1.03 on a scale from 0 to 6).

**Fig. 2:**
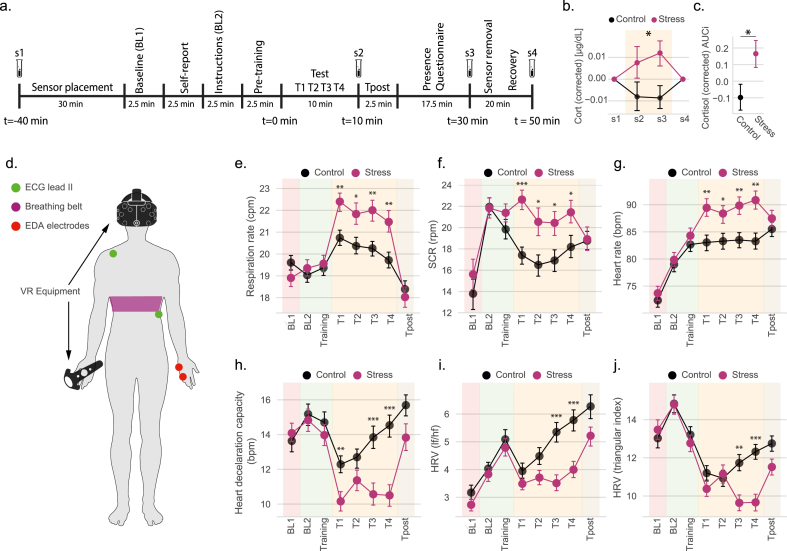
Experimental protocol applied to the IMVEST's validation and characterization study and main physiological outcomes. (**a**) Experimental protocol applied to each participant, including the different experimental phases and saliva sampling periods for cortisol analysis (s1-s4). Representative durations for sensor placement. Cortisol results corrected for circadian influence: (**b**) across the experiment; and (**c**) area under the curve with respect to increases (AUC_i_). (**d**) Schematic depiction of sensor placement and VR apparatus. Stress effects in ANS variables related to the sympathetic response (**e**, **f**, **g**) and to the parasympathetic response (**h**, **i**, **j**). Alpha significance levels: *p < 0.05, **p < 0.01, ***p < 0.001. P values corrected for the multiple comparisons across blocks. Data samples are represented by their mean (dot) and its standard error (bars).

The cortisol response was sampled from saliva at four time points during the experimental procedure (s1-s4; see Fig. 2a) and corrected for the circadian influence (Elbau et al., 2018) (see Materials and Methods for details). As expected, the cortisol response is larger in the stress group [Fig. 2b; repeated measures ANOVA (rmANOVA) group vs. timepoints (s2 and s3), group effect: F_1,116_ = 5.25, p = 0.024, ƞ_p_^2^ = 0.043]. Total cortisol (s2 and s3), computed by the area under the curve with respect to increase (AUC_i_) (Pruessner et al., 2003), is also significantly larger in the stress than in the control group [Fig. 2c; *t*(116) = 2.30, p = 0.012, Cohen's d = 0.423]. Uncorrected cortisol values can be consulted in Appendix 1-Fig. S2.

ANS variables were computed from each participant's ECG, EDA and respiration (see Fig. 2d for sensor placement) for a 2.5 baseline block (BL1) and for each of the 2.5 min experimental blocks (BL2, Training, T1-T4, Tpost; Fig. 1, Fig. 2a). As compared to the control scenario, stress exposure (blocks T1-T4) induced significant changes in variables measuring the sympathetic and parasympathetic branches of the ANS. Specifically, activation of the sympathetic branch of ANS [measured by respiration cycles per min (rpm; Fig. 2e), number of skin conductance responses (SCR) per min (Fig. 2f) and heart beats per min (bpm; Fig. 2g)] was significantly larger in the stress than in the control group during the entire test (T1-T4; all p < 0.033). Parasympathetic activity [measured by heart deceleration capacity (Fig. 2h), ratio of low and high frequency components of heart rate variability (HRV) (Fig. 2i), and the triangular index of the HRV (Fig. 2j)] was lower in the stress group, and mainly during the second half of the stress test (T3-T4) with the exception the heart deceleration capacity, which was also lower at T1 (all p < 0.007). All statistical validations for the effects shown in Fig. 2e–j can be found in Appendix 1-Table 4 and Appendix 1-Table 5.

**Fig. 3:**
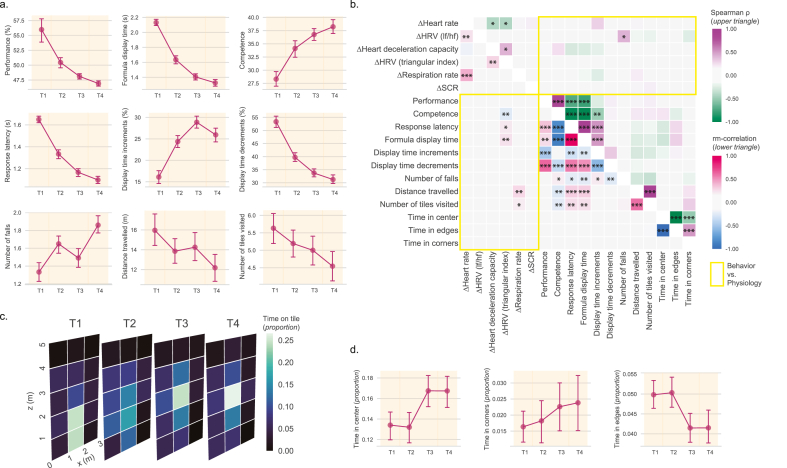
Characterization of the behavioral changes during participants' IMVEST exposure and their association with physiological variables. (**a**) Selected behavioral features and computations of ‘difficulty adjustments’ across blocks T1-T4. Data samples are represented by their mean (dot) and its standard error (bars). (**b**) Correlations between behaviors and key physiological markers. Spearman correlation coefficients between the averaged variables across T1 to T4 are shown in the upper triangular half of the matrix; repeated-measures correlation (rm-correlation) throughout T1 to T4 are shown in the lower triangular half of the matrix. Effects between physiology and behavior are highlighted in yellow. Alpha significance levels: *p < 0.05, **p < 0.01, ***p < 0.001. P values are corrected for the multiple comparisons between variables for each correlation metric. (**c**) Position heatmap for each block in the 5 by 3 tiles virtual room of IMVEST. (**d**) Position preference for different parts of the virtual room: center (3 central tiles between 1 and 4 m in the z coordinate and 1 and 2 m in the x coordinate); tiles at the corner of the room; edges (all tiles except central ones). Data samples are represented by their mean (dot) and its standard error (bars). (For interpretation of the references to colour in this figure legend, the reader is referred to the Web version of this article.)

Behavioral parameters and difficulty adjustments in the stress group (see Materials and Methods for detail on the variables analyzed) change over time (Fig. 3a; see Appendix 1-Table 6 for corresponding statistics). Performance decreases, as expected due to the difficulty titration, into values between 40% and 60%, alongside with the time the formula is displayed on the HUD. Participant's competence in the task actually increases with time, while response latency decreases, suggesting a learning effect. Display time adjustments for difficulty titration also change throughout the test. While the test progresses, the amount of adjustments to prolong the display time (increments) increase while the amount of adjustments to shorten the display time (decrements) decrease. Furthermore, there are more display time decrements than increments which suggests that difficulty needs to be increased for most participants, particularly in the first trials. Regarding movement, falls increase while distance decreases as time goes on. Due to the programmed 5% chance that a successful trial is processed as incorrect, from an average of 149 completed trials across the test, each participant had on average 7 falsely incorrect trials. In the post experimental debriefing, some participants reported they had noticed the unexpected negative feedback, but were unsure about its interpretation and continued engaged in the task.

Due to the inter – and intra-individual variability in behaviors, it is important to assess whether there is a relationship between these variables and the physiological activation. To this end, we computed two different correlational assessments between behavioral variables depicted in Fig. 3a and the physiology variables (changes from baseline BL1, Δ prefix variables) represented in Fig. 2e–j (correction for multiple comparisons applied for analyses within each correlation modality): i) Spearman rank correlation coefficient to assess inter-individual associations for variables averaged across T1-T4; and ii) repeated measures correlation (rm-correlation) to assess intra-individual associations of changes over time. As can be seen in Fig. 3b (correlation scores and p-values, corrected and uncorrected, can be seen in Appendix 1-Table 7), and supporting that physiological changes are not determined by behavioral components, behavioral and physiological variables tend not to correlate, with very few exceptions. Indeed, for Spearman rank correlations, there was only a moderate positive correlation between the number of falls and the HRV parameter representing the low/high frequency ratio. Regarding intra-individual associations across T1-T4 measured by rm-correlations, there were expected associations between: i) changes in exploration (i.e., distance travelled, and number of tiles visited) and those observed in respiration rate; and ii) changes in task competence (and the consequent reductions in response time and formula display time) and those observed in a marker for HRV (delta-HRV triangular index). Furthermore, to confirm that the display time adjustments are important to keep physiological activation high, we computed an additional variable (see Methods for details) by subtracting the proportion of time increments (made when participant's performance is insufficient, to allow for performance improvement) from the proportion of decrements (made when participant's performance is better than the targeted criterion, to allow for performance impairment) (Appendix 1-Fig. S3a) and classified our subjects in the stress group into quartiles based on this variable (Appendix 1-Fig. S3b). As can be seen in Appendix 1-Fig. S3c-h, quartiles have similar values of physiological activation (all n.s.; see Appendix 1-Table 8 for statistical tests and analyses).

Due to the emerging importance of locomotion and navigation in virtual environments for the prediction of stress markers (Rodrigues et al., 2020), we also measured participants positioning in the stress environment. We observed a change in positioning from the first half to the second half of the tests (Fig. 3c and d; see Appendix 1-Table 6 for corresponding statistics), but note that these changes were not associated with stress physiology (Fig. 3b). Regarding salivary cortisol, there were no significant correlations between total cortisol and any of the behavioral, adjustment variables and movement variables (all p > 0.278; see Appendix 1-Table 9). Collectively, these data and analyses indicate that IMVEST induces changes in key components of the ANS and salivary cortisol regardless of how participants behaved or how difficulty was titrated.

#### 3.2.2 A classifier confirms efficiency of IMVEST to trigger physiological stress responses

To understand how IMVEST elicits changes in the ANS, we assessed which out of 50 ANS variables [25 absolute value variables and 25 change from baseline BL1 variables (Δ prefix); see Materials and Methods and Table 2 for their description] significantly differ between control and stress (independent samples t-tests for each variable). We performed this analysis for each test block and, then, computed the proportion of variables in which we found significant differences. This analysis had two purposes: i) understanding which relevant variables signal stress over time; and ii) interrogating whether the key manipulation of negative feedback implemented in the transition from T2 to T3 was effective in further fueling physiological stress activation.

**Fig. 4:**
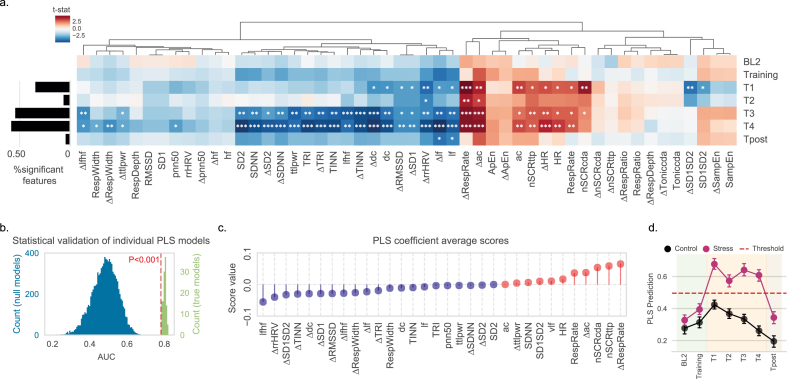
Characterization of the autonomic response to IMVEST and development of a PLS model. (**a**) Statistic heatmap for independent samples *t*-test (stress vs. control) for each ANS variable (see Table 2 for variable description). Variables are grouped together with hierarchical clustering according to the similarities in the *t*-test test statistics to facilitate interpretation. A clear distinction between stress and control can be observed at T1, T3 and T4 indicating parasympathetic withdrawal and sympathetic activation, with more than 50% of the tested variables showing significant differences at T3 and T4. Alpha significance levels: *p < 0.05, **p < 0.01, ***p < 0.001. P values corrected for multiple comparisons between variables in each block. (**b**) Statistical validation of each individual PLS model by non-parametrically testing the significance of their area under the curve (AUC). Individual models' AUC are compared to the AUC distribution of 10 k models trained on datasets with randomly shuffled labels at the participant level. (**c**) Average of the PLS coefficient scores across all individual models, informing about the magnitude and direction of the influence that each variable exerts in the model's prediction score. (**d**) Model prediction scores for both stress and control group. Average of each model's optimal decision threshold represented by the dashed line. Data samples are represented by their mean (dot) and its standard error (bars).

As shown in Fig. 4a, significant differences between stress and control are only present during T1-T4 and Tpost. These changes are consistent with stress induced changes in ANS responses, with a clear pattern of elevated HR, respiration rate and SCR, and a more pronounced decrease in HRV variables in the second half of the stress test (i.e., T3-T4) (Fig. 3a). Importantly, there was a decline in ANS differences between control and stress groups in T2, supporting a habituation of individuals to the impinged challenges, while there were again marked group differences for a large number of variables for blocks T3 and T4. The latter supports the efficiency of the negative feedback manipulation introduced in the transition to T3.

Then, we used a classifier based on partial-least-squares regression (PLSR) with feature selection, chosen for being robust in the presence of colinear variables (see Materials and Methods for details), in order to develop a model that discriminates between stress and control participants using ANS variables. We used a leave-one-out (LOO) procedure where, for each participant, a PLSR model was trained with all the remaining participants. This way, the predictions for any given participant always originate from a model that did not learn from this participant's data. We used all stress blocks for PLSR training, except block T2 due to the low number of discriminatory features present in this bloc as shown in Fig. 4a. Using optimal thresholding (see Materials and Methods for details), our model predictions identified participants in the stress group from an average area under the curve (AUC) of 0.80 (Fig. 4b). The ANS variables that were most relevant for the individual models were based on respiration, SCR and HRV (the contribution of these variables to the model is depicted in Fig. 4c). The average value of the stress prediction score can be seen in Fig. 4d for all blocks and for both stress and control groups. As expected, scores were higher for the stress group during test blocks and, interestingly, there is an initial elevation in the control group that then attenuates over time. Furthermore, as the model was trained with test blocks T1, T3 and T4, these scores were low in the remaining blocks (i.e., BL2, Training and Tpost). Finally, and given that individual variation is an important feature of psychosocial stressors (Miller and Kirschbaum, 2019), we examined individual variation in ANS changes across experimental blocks in both the control and the stress group using the PLSR model as a stress predictor. As expected, more blocks were classified as *stress* in the experimental group (Appendix 1-Fig. S4) and the higher this number, the larger the cortisol response (Appendix 1-Fig. S5).

## 4 Discussion

IMVEST is a new stress induction method for humans for full implementation in VR that can be easily employed using a head mounted display (HMD) and a room of standard size and characteristics. It capitalizes on the multimodal capabilities of VR, that allow participants to be fully immersed and navigate in environments enriched with simultaneous stressful elements. A key characteristic that goes beyond current state of the art on VR stress procedures based on motivated performance is a continuous adjustment of the difficulty inflicted by the mathematical challenges to the individual's performance in real time. Importantly, we validate here the effectiveness of this manipulation to lead to changes in physiological parameters regardless of performance levels (see Appendix 1-Table 10 for a comparison of our results with other stress protocols). Furthermore, the test provides an additional control scenario offering a comparable but not stressful situation. The scripted nature of IMVEST allows its flexible adaptation to laboratories with different languages and targeted samples, while the programmed adjustment of difficulty provides consistency in the stressor applied at the individual level. Importantly, as the software records a log of the participant's positioning and choices, IMVEST allows implementing detailed behavioral analysis.

IMVEST contains key stress components, such as the simultaneously exposure to mental and environmental challenges, along with intense visual and auditory stimulation. In addition, IMVEST incorporates important elements for effective stress induction (Dickerson and Kemeny, 2004), including components of uncontrollability and unpredictability, social-evaluative threat, and threat to other central goals (such as exposure to dangerous explosions and possibility to fall). Stressor controllability and predictability play a crucial role in the triggering of stress responses (Mineka and Hendersen, 1985). In IMVEST, lack of controllability and predictability are introduced in several ways: in a percentage of trials, the computer compatibilized correct performance as incorrect, introducing a bewildering component; the tiles breaking in the floor under failure were chosen at random, and therefore participants could not control the probability to fall through their spatial location in the virtual environment. Social-evaluative threat relates to situations in which the individual, or ‘self’, could be negatively assessed by others (Baumeister and Leary, 1995) and negative social comparison causes strong distress (Andrews et al., 2007; Woody et al., 2018). In IMVEST, social-evaluative threat is implemented through the continuous written information that participant's performance lays behind that of the group's average, as well as by several oral messages that prompt the participant to improve their performance. This manipulation was implemented as competition can increase stress responses (Shiban et al., 2016). In addition, IMVEST includes components of threat to other individual's central goals, such as own's physical integrity, given that the virtual threat imposed by the scenario contains elements that would be highly dangerous in the real world, such as explosions and floor breaking. In addition, and following evidence that multimodal stressors are highly impactful (Weger et al., 2020) and certainly more effective than unimodal ones (Maras et al., 2014), our stress procedure contains a combination of sensory stressors, including audio (explosion noise; loud stressful music) and visual (explosion components) effects.

Its application to 118 participants demonstrated IMVEST efficiency to activate the two key stress physiological systems, leading to increases in cortisol and alterations in a broad range of parameters involving the functioning of the two ANS branches, SNS and PNS. In addition, given that negative feedback about own's performance is an important stressor (Kassam et al., 2009), we added a strong manipulation combining negative feedback and social-evaluative threat in the middle of the test, at the transition from T2 to T3 blocks (participants were told that their performance so far was insufficient and the task needed to be re-started). We validated the effectiveness of this manipulation as an important element to correct for a natural habituation to the given challenges and ensuring an enduring stress activation.

IMVEST takes advantage of the proven effectiveness of the inclusion of socio-evaluative threat in previous laboratory stress procedures to boost stress reactivity [e.g., SECPT (Schwabe et al., 2008), MAST (Smeets, 2011) and the TSST (Kirschbaum et al., 1993)] beyond similar versions of the same tests without such component. It also builds on recent developments of stress tasks on VR, as this approach removes variability related to experimental differences across laboratories. However, previous work has indicated that TSST-VR versions based on public speaking were highly dependent on technology, as they include human avatars for which graphical fidelity is still challenging in VR, and earlier VR technologies frequently induced dizziness in participants (Helminen et al., 2019). IMVEST presents a clear advantage over VR stress tests based on the public speaking task as it does not require interaction with human avatars, which makes our test more robust for replicability and realism. Moreover, IMVEST relies on well-developed features in VR technology to deliver stress. Navigation in VR can now be done easily without disruptive motion sickness, due to low latencies and stability (Chénéchal and Goldman, 2018). Current HMD screen resolutions allow text and calculations to be projected and readable in the HUD field of vision, while still allowing visualization of the surrounding environment. Necessary sounds, graphics and visual effects for this test are simple and can be reliably reproduced in VR.

Furthermore, inspired by the 2D-screen delivered MIST test for neuroimaging (Dedovic et al., 2005), IMVEST adjusts difficulty to individuals' performance in the arithmetic task, a notable advancement to ensure that observed differences in stress reactivity are not due to differences in mathematical competence but to individuals’ stress reactivity. Importantly, our results validate the effectiveness of this manipulation.

Another important feature of IMVEST is that it allows participants to move in the virtual environment, while multi-dimensional and high-density behavioral and physiological responses can be recorded. This is an advantage over 2D-screen tests in which participants' are typically seating or laying down which prevents from obtaining information of participant's body movements and spatial navigation, and these features are emerging as highly informative of individual's vulnerability to stress (Rodrigues et al., 2020). We also found high levels of presence in our participants (Slater, 2003), a feeling that facilitates effectiveness in laboratory stress tasks (Morina et al., 2014) and that it is facilitated by immersion in 3D over 2D tests (Kober et al., 2012; Roettl and Terlutter, 2018).

IMVEST can be used for the purpose of diagnostics or research. For the former, it can help assessing whether a particular individual shows aberrant stress responses when confronted with stressful challenges. Another possible diagnostic tool could be to evaluate whether a particular treatment (e.g., mindfulness, psychotherapy or drugs) improves exacerbated stress responses in the individual or groups of treated subjects. Regarding research applications, IMVEST can be used as a means to elicit stress and investigate how it affects individual's functioning in a number of different domains (e.g., how stress exposure affects subsequent learning or decision-making capabilities) or to investigate how specific behavioral and/or (neuro)physiological parameters are affected by specific components of the stressful experience and/or how they vary across different individuals. In fact, the number of applications and analyses are numerous, given that stress can impact a myriad of physiological systems, from the brain to endocrine, cardiovascular, immune, reproductive and gastrointestinal systems.

Our study has limitations related to the validation part. Indeed, we recruited men participants aged 18-38 years-old. In the future, it will be important to characterize responses to IMVEST in other sex and age groups. In addition, all participants in our study were tested in the afternoon and it would be useful to assess the test validity for other time points. However, since the stressful narrative in IMVEST is not artificial, like in mock interview-based tests, we foresee that it could be advantageous for experimental programs where it is relevant to deliver stress to the same individual more than once. In addition, if required, IMVEST allows the implementation of additional elements, such as incentivizing performance with monetary rewards, penalizing falling with additional aversive consequences, or introduce a component of inter-subjects’ competition.

In conclusion, IMVEST represents a robust and well standardized procedure for effective acute stress induction in humans that goes beyond state-of-the-art in several aspects. Together with its ease of use with minimal personnel and standardization for controlled experiments, IMVEST's innovations represent crucial advances to ensure comparability of stressor challenge across individuals and laboratories. These advances can improve the power of stress studies involving, for example, multi-center trials, longitudinal studies or meta-analysis.

## Funding

This work was supported by grants from The 10.13039/100001275Oak Foundation, the 10.13039/100000001Swiss National Science Foundation (SNSF; NCCR Synapsy grant nos. 158776 and 185897; Sinergia no. 183564) and intramural funding from the 10.13039/501100001703EPFL to C.S. and an 10.13039/501100001711SNSF SPARK grant no. 196558 to J.R. The funders had no role in study design, data collection and analysis, decision to publish or preparation of the manuscript.

## CRediT authorship contribution statement

**João Rodrigues:** designed the experiments, performed experiments; analyzed the physiological data; analyzed the motion capture data and applied machine learning analyses, wrote the manuscript. **Erik Studer:** designed the experiments, performed experiments; analyzed the physiological data. **Stephan Streuber:** designed the experiments, performed experiments. **Carmen Sandi:** wrote the manuscript, supervised and supported the project. designed the experiments.

## Declaration of competing interest

None.

## Data Availability

Data will be made available on request.
