# Supplementary material for: IMVEST, an immersive multimodal virtual environment stress test for humans that adjusts challenge to individual's performance
Source: Neurobiol Stress. 2021 Aug 13;15:100382. doi: 10.1016/j.ynstr.2021.100382 (PMC8385118; doi:10.1016/j.ynstr.2021.100382)
Supplement: Multimedia component 1 [file mmc1.pdf]

Appendix 1

IMVEST, an immersive multimodal virtual environment stress test for humans that adjusts challenge to individual's performance

João Rodrigues, Erik Studer, Stephan Streuber, Carmen Sandi

Contents

Detailed statistical analysis........ 3
Statistical software........ 3
Supplementary Tables........ 4
Appendix 1-Table 1........ 4
Appendix 1-Table 2........ 5
Appendix 1-Table 3........ 5
Appendix 1-Table 4........ 6
Appendix 1-Table 5........ 7
Appendix 1-Table 6........ 8
Appendix 1-Table 7........ 9
Appendix 1-Table 8........ 15
Appendix 1-Table 9........ 16
Appendix 1-Table 10 17
Supplementary Figures 18
Figure S1. 18
Figure S2 19
Figure S3 20
Figure S4. 21
Figure S5. 21
Appendix 1-Algorithm S1 22
References........ 23

Detailed statistical analysis

rmANOVA was used for each analysis in Figures 2e-j, followed by post-hoc t-tests if the interaction term was significant. Sphericity assumption violations were tested with the Mauchly's test of sphericity and Greenhouse-Geisser corrections were applied whenever assumptions were violated. Pairwise comparisons of demographics, personality traits, anxiety and presence between the two groups were done with t-tests or Mann-Whitney U test if a Shapiro-Wilk normality test suggests a violation of the assumption of normality. One-sided t-tests were used whenever the direction of the effect was anticipated, as in Figure2c, and Figures 2e-j. Before these t-tests, both normality and homogeneity of variances were assessed, respectively, with the Shapiro-Wilk and Levene's test. When these tests warned about violations of normality and homogeneity of variances, the Mann-Whitney U test or the Welch's t-test were also performed. Two-sided t-tests were used for the comparisons in Figure 4a. Correction for multiple comparisons was applied with the Benjamini & Hochberg procedure for controlling the false discovery rate, correcting for comparing results within 8 blocks in Figures 2e-j, 144 pairwise Spearman correlations in Figure 3b, 144 rm-correlations in Figure 3b, 50 features (per block) in Figure 4a, 4 tests for presence variables, 3 tests for pre-test variables (PANAS and STAI-T) and 12 pairwise Spearman correlations between cortisol and behavioral variables.

Statistical software

Statistical analyses were performed using Jamovi v1.6.15.0 (Mann-Whitney U test for Table 1, t-tests for cortisol analysis in Figure 2c, t-tests and Mann-Whitney U test for presence variables, rmANOVA for cortisol in Figure 2b, physiology in Figures 2e-j and respective post-hoc tests; rmANOVA for behavioral variables in Figures 3a and 3d), Python v3.7.1 (t-tests in Figure 4a with SciPy v1.6.1, Spearman correlations in Figure 3b with SciPy v1.6.1 and rm-correlations in Figure 3b with Pingouin v0.3.10). Outliers were identified and replaced using the MATLAB function *filloutliers* with default options.

Supplementary Tables

Appendix 1-Table 1: settings used for the Cardiovascular Signal Toolbox (changes from default).

**Parameter**	**Value**
Global Settings	
Fs (Hz)	1000
Window length (s)	150
Rejection threshold	0.8
SQI Analysis Settings	
Low quality threshold	0.3
AF detection settings and PVC detection	
AF ON	0 (false)
Frequency domain analysis settings	
ULF (Hz)	Not used
VLF (Hz)	0.0033 to 0.04 (default)
LF (Hz)	0.04 to 0.15 (default)
HF (Hz)	0.15 to 0.4 (default)
SDANN and SDNNI analysis settings	
SD segment length (s)	150
Entropy settings	
MSE ON	0 (false)
DFA ON	0 (false)
Heart rate turbulence (HRT) – settings	
HRT ON	0 (false)

Appendix 1-Table 2: State variables before the test. Comparisons with Shapiro-Wilk p-value < 0.05 are done using the Mann-Whitney U test.

	Group	N	Mean	Standard deviation	Shapiro-Wilk p-value	T-statistic	Mann-Whitney U	p-value	p-value corrected
State anxiety	Control	61	33.50	6.67	0.081	-1.93		0.056	0.167
	Stress	57	31.20	6.20					
Positive affect	Control	61	29.20	4.12	0.001		1629	0.556	0.633
	Stress	57	29.40	4.54					
Negative affect	Control	61	24.10	4.51	0.007		1650	0.633	0. 633
	Stress	57	23.80	4.61					

Appendix 1-Table 3: Presence variables after the test. Comparisons with Shapiro-Wilk p-value < 0.05 are done using the Mann-Whitney U test.

	Group	N	Mean	Standard deviation	Shapiro-Wilk p-value	T-statistic	Mann-Whitney U	p-value	p-value corrected
General presence	Control	61	5.21	0.78	1.640e-7		1288	0.068	0.270
	Stress	52	4.87	1.03					
Spatial presence	Control	61	4.13	0.72	0.002		1454	0.445	0.790
	Stress	52	4.01	0.69					
Involvement	Control	61	3.41	0.68	0.097	-0.267		0.790	0.790
	Stress	52	3.37	0.74					
Experienced realism	Control	61	2.59	0.78	0.078	0.480		0.632	0.790
	Stress	52	2.65	0.61					

Appendix 1-Table 4: Statistical tests for Figures 2e-j. Repeated measures ANOVA (2 by 8), within-subjects' effects, for the Group (stress, control) x Block (BL1, BL2, Training, T1, T2, T3, T4, Tpost) model design, to predict each one of the dependent variables in Figures 2e-j. Greenhouse-Geisser sphericity correction applied to all tests due to sphericity violations, as seen from the Mauchly's test p-values.

Dependent variable	Mauchly's test p-value	Independent variables	Sum of squares	df	Mean square	F-statistic	p-value	η^2_p
Respiration rate (cpm)	5.102e-27	Block	1175	4.91	239.57	42.08	2.413e-36	0.266
		Block*Group	211	4.91	43.08	7.57	8.327e0-7	0.061
		Residual	3240	569.10	5.69			
SCR (rpm)	3.795e-22	Block	3691	4.70	785.0	11.62	3.3376e-10	0.091
		Block*Group	742	4.70	157.9	2.34	0.044479	0.020
		Residual	36866	545.53	67.6			
Heart rate (bpm)	1.629e-65	Block	20148	3.13	6436.7	162.6	1.2859e-68	0.584
		Block*Group	1531	3.13	489.0	12.4	6.1136e0-8	0.096
		Residual	14373	363.11	39.6			
Heart deceleration capacity (bpm)	5.311e-6	Block	1664	5.94	280.0	23.47	4.0881e-25	0.168
		Block*Group	461	5.94	77.6	6.50	1.1984e0-6	0.053
		Residual	8225	689.53	11.9			
HRV (lf/hf)	3.998e-11	Block	611.5	5.63	108.54	25.28	1.0222e-25	0.179
		Block*Group	86.9	5.63	15.43	3.59	0.0020977	0.030
		Residual	2805.7	653.59	4.29			
HRV (triangular index)	6.262e-10	Block	1837	5.62	326.70	47.21	1.0027e-45	0.289
		Block*Group	254	5.62	45.10	6.52	2.0889e0-6	0.053
		Residual	4512	652.14	6.92			
Note. Type 3 Sums of Squares								

Appendix 1-Table 5: Statistical post-hoc tests for Figures 2e-j. Independent samples Student's t-tests for each independent variable in Figures 2e-j at each Block (BL1, BL2, Training, T1, T2, T3, T4, Tpost). Violation of assumptions of normality and equal variance are tested with the Shapiro-Wilk and the Levene's tests respectively. In case of normality assumption violation, the Mann-Whitney (MW) U test is performed and its p-value is presented. In case of equal variance assumption violation, the Welch's t-test is performed and its p-value presented. The Benjamini-Hochberg (BH) procedure was performed to decrease the false discovery rate by adjusting the p-values for the 8n block comparisons.

	Block	T-Statistic	df	p-value	p-value BH corrected	Cohen's d	Equal variance or normality assumption violation	
							**MW p-value**	**Welch p-value**
**Respiration rate (cpm). H_a stress > control**	BL1	-1.373	116	0.9137949	0.913795	0.2529		
	BL2	0.615	116	0.2698035	0.431686	0.1133		
	Training	0.365	116	0.3578009	0.477068	0.0673		
	T1	2.978	116	0.0017692	0.005611	0.5485		
	T2	2.370	116	0.0097218	0.019444	0.4366		0.0102676
	T3	2.920	116	0.0021040	0.005611	0.5379		
	T4	2.974	116	0.0017887	0.005611	0.5479		
	Tpost	-0.576	116	0.7169977	0.819426	0.1060		
**SCR (rpm). H_a stress > control**	BL1	0.8777	116	0.1909554	0.2546072	0.1617	0.1601304	
	BL2	-0.0990	116	0.5393361	0.5393361	0.0182		
	Training	1.2752	116	0.1023855	0.1638168	0.2349		
	T1	4.2340	116	2.3092e-5	0.0001847	0.7800		
	T2	2.5450	116	0.0061201	0.0244804	0.4688		
	T3	2.2856	116	0.0120468	0.0321248	0.4211		
	T4	2.1486	116	0.0168729	0.0337458	0.3958		
	Tpost	0.0984	116	0.4608727	0.5267117	0.0181		
**Heart rate (bpm). H_a stress > control**	BL1	0.730	116	0.2335092	0.2668677	0.1344	0.2590605	
	BL2	0.434	116	0.3326956	0.3326956	0.0799		
	Training	0.843	116	0.2004558	0.2668677	0.1553		
	T1	2.939	116	0.0019874	0.0057459	0.5414		
	T2	2.315	116	0.0111731	0.0223462	0.4266		
	T3	2.912	116	0.0021547	0.0057459	0.5364	0.0028664	
	T4	3.434	116	4.1320e-4	0.0033056	0.6326	4.0450e-4	
	Tpost	0.960	116	0.1694102	0.2668677	0.1769		
**Heart deceleration capacity (bpm). H_a stress < control**	BL1	0.556	116	0.7104800	0.71048	0.1025		
	BL2	-0.392	116	0.3478982	0.3975979	0.0722		
	Training	-0.824	116	0.2058729	0.2744972	0.1518		
	T1	-2.860	116	0.0025091	0.0066909	0.5270	0.0010906	
	T2	-1.618	116	0.0541478	0.0866365	0.2981		
	T3	-3.587	116	2.4558e-4	0.0009823	0.6608		
	T4	-4.285	116	1.8931e-5	0.0001514	0.7895		
	Tpost	-1.907	116	0.0294986	0.0589972	0.3513	0.0071360	
**HRV (lf/hf). H_a stress < control**	BL1	-1.371	116	0.086490	0.138384	0.253	0.105745	0.085273
	BL2	-0.567	116	0.285952	0.285952	0.104	0.184941	
	Training	-0.631	116	0.264598	0.285952	0.116	0.339179	
	T1	-1.215	116	0.113328	0.151104	0.224	0.241916	0.110979
	T2	-1.858	116	0.032885	0.06577	0.342	0.061411	
	T3	-4.061	116	4.4493e-5	0.0003559	0.748	4.2557e-5	
	T4	-3.667	116	1.8563e-4	0.0007425	0.676	8.7719e-4	1.6344e-4
	Tpost	-1.919	116	0.028742	0.06577	0.353	0.044362	0.027690
**HRV (triangular index). H_a stress < control**	BL1	0.6409	116	0.738574	0.738574	0.1181	0.744423	
	BL2	-0.0568	116	0.477419	0.6365587	0.0105		
	Training	-0.6926	116	0.244964	0.3919424	0.1276	0.237745	
	T1	-1.5283	116	0.064577	0.129154	0.2815		
	T2	0.3477	116	0.635634	0.7264389	0.0640	0.546089	
	T3	-3.4942	116	3.3705e-4	0.0013482	0.6437		
	T4	-4.4451	116	1.0110e-5	8.09E-05	0.8189		
	Tpost	-2.1030	116	0.018816	0.050176	0.3874		

Appendix 1-Table 6: Statistical tests for Figures 2e-j. Repeated measures ANOVA, within-subjects' effects for the Block (T1, T2, T3, T4, Tpost), to predict each one of the dependent variables in Figures 3a and 3d. Greenhouse-Geisser sphericity correction applied to tests where sphericity violations were detected, as seen from the Mauchly's test p-values.

Dependent variable	Mauchly's test p-value	Independent variables	Sum of squares	df	Mean square	F-statistic	p-value	η^2_p
Performance (%)	1.6112e-51	Block	2958	1.18	2501.4	25.1	9.2069e-7	0.285
		Residual	7426	74.51	99.7			
Formula display time (%)	2.939e-4	Block	25.78	2.41	10.6986	197	4.9881e-47	0.757
		Residual	8.26	151.83	0.0544			
Competence	2.018e-7	Block	4064	2.10	1939.4	36.4	9.8657e-14	0.366
		Residual	7025	132.00	53.2			
Response latency (s)	2.018e-7	Block	11.99	2.45	4.9005	196	1.1830e-47	0.757
		Residual	3.85	154.16	0.0250			
Display time increments (%)	0.110	Block	0.540	3	0.1798	13.7	4.0610e-8	0.178
		Residual	2.483	189	0.0131			
Display time decrements (%)	1.826e-5	Block	2.27	2.22	1.0214	33.0	2.3027e-13	0.344
		Residual	4.33	139.96	0.0309			
Number of falls	0.056	Block	10.7	3	3.566	6.21	4.8062e-4	0.090
		Residual	108.6	189	0.574			
Distance traveled (m)	5.314e-4	Block	340	2.41	141.3	6.30	0.0012130	0.091
		Residual	3400	151.52	22.4			
Number of tiles visited	0.890	Block	25.6	3	8.54	2.55	0.056862	0.039
		Residual	632.4	189	3.35			
Time in center (proportion)	0.005	Block	0.0675	2.52	0.02677	2.98	0.041904	0.051
		Residual	1.2663	141.14	0.00897			
Time in corners (proportion)	1.696e-4	Block	0.00216	2.26	9.55e-4	0.765	0.48180	0.013
		Residual	0.15787	126.53	0.00125			
Time in edges (proportion)	0.005	Block	0.0675	2.52	0.02677	2.98	0.041904	0.051
		Residual	1.2663	141.14	0.00897			
Note. Type 3 Sums of Squares								

Appendix 1-Table 7: Correlations for Figure 3b.

Variable name	Variable name	rm-correlation	p-value	p-value BH corrected	Spearman correlation	p-value	p-value BH corrected
ΔHRV (lf/hf)	ΔHeart rate	0.237785	0.001684	0.008884	0.076679	0.570767	0.747846
ΔHeart rate deceleration capacity	ΔHeart rate	-0.187865	0.013592	0.059415	-0.404848	0.001786	0.016071
ΔHeart rate deceleration capacity	ΔHRV (lf/hf)	0.052183	0.496599	0.893879	-0.080957	0.549405	0.743884
ΔHRV (triangular index)	ΔHeart rate	-0.070274	0.359644	0.737671	-0.413015	0.001408	0.014363
ΔHRV (triangular index)	ΔHRV (lf/hf)	0.020436	0.790172	1	-0.002398	0.985874	0.990496
ΔHRV (triangular index)	ΔHeart rate deceleration capacity	0.286352	0.00014	0.00126	0.410293	0.001525	0.014585
ΔRespiration rate	ΔHeart rate	0.406447	0	0	0.16846	0.210336	0.543197
ΔRespiration rate	ΔHRV (lf/hf)	0.134614	0.078308	0.272298	-0.007778	0.954208	0.990496
ΔRespiration rate	ΔHeart rate deceleration capacity	-0.097921	0.201272	0.504831	-0.091003	0.500796	0.696562
ΔRespiration rate	ΔHRV (triangular index)	-0.105421	0.168715	0.452866	-0.149144	0.268175	0.603393
ΔSCRcda	ΔHeart rate	-0.074807	0.32941	0.717837	0.179933	0.180465	0.535886
ΔSCRcda	ΔHRV (lf/hf)	-0.006229	0.935361	1	-0.101504	0.452471	0.694069
ΔSCRcda	ΔHeart rate deceleration capacity	0.037513	0.625151	1	-0.017825	0.895299	0.978433
ΔSCRcda	ΔHRV (triangular index)	-0.024492	0.749786	1	0.033381	0.805291	0.940531
ΔSCRcda	ΔRespiration rate	0.06637	0.387017	0.769007	-0.095411	0.480194	0.694069
Performance	ΔHeart rate	-0.032134	0.675602	1	-0.109411	0.41784	0.694069
Performance	ΔHRV (lf/hf)	-0.139602	0.06778	0.24241	-0.103124	0.445249	0.694069
Performance	ΔHeart rate deceleration capacity	-0.058382	0.44681	0.833681	0.160034	0.234384	0.58788
Performance	ΔHRV (triangular index)	-0.011561	0.880358	1	0.097355	0.471249	0.694069
Performance	ΔRespiration rate	0.07282	0.342454	0.717837	0.03027	0.823132	0.946911
Performance	ΔSCRcda	0.064567	0.400074	0.77506	-0.095929	0.4778	0.694069
Competence	ΔHeart rate	0.112477	0.141826	0.401839	-0.108439	0.422014	0.694069
Competence	ΔHRV (lf/hf)	0.029027	0.705435	1	-0.105458	0.434962	0.694069
Competence	ΔHeart rate deceleration capacity	-0.076882	0.316136	0.711306	0.254472	0.056103	0.286123
Competence	ΔHRV (triangular index)	-0.244989	0.001199	0.007645	0.140264	0.29803	0.618634

Appendix 1-Table 7 (continued): Correlations for Figure 3b.

Variable name	Variable name	rm-correlation	p-value	p-value BH corrected	Spearman correlation	p-value	p-value BH corrected
Competence	ΔRespiration rate	-0.046443	0.545189	0.937234	0.140394	0.297579	0.618634
Competence	ΔSCRcda	-0.041428	0.589481	0.980333	-0.110643	0.412587	0.694069
Competence	Performance	0.099533	0.19392	0.502878	0.864791	0	0
Response latency	ΔHeart rate	-0.113964	0.136599	0.395774	0.131514	0.329483	0.656758
Response latency	ΔHRV (lf/hf)	-0.09834	0.199344	0.504831	0.068577	0.612247	0.761994
Response latency	ΔHeart rate deceleration capacity	0.031112	0.685364	1	-0.277612	0.036548	0.223672
Response latency	ΔHRV (triangular index)	0.217622	0.004134	0.020405	-0.074605	0.581261	0.747846
Response latency	ΔRespiration rate	0.14956	0.050209	0.192049	-0.146617	0.276462	0.604267
Response latency	ΔSCRcda	0.069696	0.363619	0.737671	0.140135	0.298481	0.618634
Response latency	Performance	0.351367	0.000002	0.000027	-0.70936	0	0
Response latency	Competence	-0.81627	0	0	-0.93784	0	0
Formula display time	ΔHeart rate	-0.120506	0.115339	0.367643	0.084781	0.530638	0.72489
Formula display time	ΔHRV (lf/hf)	-0.078752	0.304475	0.705828	0.099819	0.46005	0.694069
Formula display time	ΔHeart rate deceleration capacity	0.028958	0.706105	1	-0.250972	0.059689	0.294595
Formula display time	ΔHRV (triangular index)	0.241664	0.001404	0.008117	-0.105976	0.432694	0.694069
Formula display time	ΔRespiration rate	0.124573	0.103483	0.337712	-0.156469	0.245106	0.603393
Formula display time	ΔSCRcda	0.057638	0.452636	0.834377	0.110837	0.411761	0.694069
Formula display time	Performance	0.265538	0.000431	0.003468	-0.770936	0	0
Formula display time	Competence	-0.884511	0	0	-0.970703	0	0
Formula display time	Response latency	0.959872	0	0	0.95936	0	0
Display time increments	ΔHeart rate	0.061049	0.426288	0.80521	0.308206	0.019677	0.143363
Display time increments	ΔHRV (lf/hf)	0.043065	0.574842	0.966493	0.095346	0.480493	0.694069
Display time increments	ΔHeart rate deceleration capacity	0.06273	0.413641	0.791089	-0.217656	0.103859	0.39726
Display time increments	ΔHRV (triangular index)	0.03025	0.693636	1	-0.112717	0.403826	0.694069
Display time increments	ΔRespiration rate	-0.165397	0.030136	0.124616	-0.2395	0.072759	0.318063
Display time increments	ΔSCRcda	-0.03371	0.660656	1	0.151348	0.261086	0.603393

Appendix 1-Table 7 (continued): Correlations for Figure 3b.

Variable name	Variable name	rm-correlation	p-value	p-value BH corrected	Spearman correlation	p-value	p-value BH corrected
Display time increments	Performance	-0.475051	0	0	-0.248315	0.062534	0.298991
Display time increments	Competence	0.031198	0.684539	1	-0.478351	0.000168	0.001972
Display time increments	Response latency	-0.317093	0.000023	0.000246	0.522297	0.000031	0.000524
Display time increments	Formula display time	-0.260894	0.000547	0.003984	0.506935	0.000057	0.000871
Display time decrements	ΔHeart rate	-0.084691	0.269336	0.633975	0.103578	0.443238	0.694069
Display time decrements	ΔHRV (lf/hf)	-0.030424	0.691965	1	0.068512	0.612584	0.761994
Display time decrements	ΔHeart rate deceleration capacity	-0.06929	0.366425	0.737671	-0.027029	0.84181	0.954052
Display time decrements	ΔHRV (triangular index)	0.072814	0.342497	0.717837	0.098393	0.466516	0.694069
Display time decrements	ΔRespiration rate	0.117798	0.123804	0.386571	-0.155172	0.249087	0.603393
Display time decrements	ΔSCRcda	0.031929	0.677562	1	0.097744	0.469471	0.694069
Display time decrements	Performance	0.618719	0	0	0.187257	0.163073	0.535886
Display time decrements	Competence	-0.359718	0.000001	0.000016	0.178701	0.183516	0.535886
Display time decrements	Response latency	0.55631	0	0	-0.152061	0.25882	0.603393
Display time decrements	Formula display time	0.501258	0	0	-0.147394	0.273894	0.604267
Display time decrements	Display time increments	-0.583256	0	0	0.330049	0.012166	0.097965
Number of falls	ΔHeart rate	0.004734	0.950859	1	0.001763	0.989617	0.990496
Number of falls	ΔHRV (lf/hf)	0.008656	0.910267	1	0.41602	0.001288	0.014081
Number of falls	ΔHeart rate deceleration capacity	0.048451	0.527931	0.917881	0.101178	0.453931	0.694069
Number of falls	ΔHRV (triangular index)	-0.010573	0.89051	1	0.111123	0.410551	0.694069
Number of falls	ΔRespiration rate	-0.05303	0.48963	0.891827	-0.139926	0.299209	0.618634
Number of falls	ΔSCRcda	-0.104401	0.172897	0.456091	0.121733	0.367024	0.676563
Number of falls	Performance	-0.153735	0.044062	0.17286	-0.041642	0.758419	0.906548
Number of falls	Competence	0.199911	0.008557	0.039671	-0.034657	0.798	0.939185
Number of falls	Response latency	-0.233747	0.002028	0.010344	0.008149	0.952028	0.990496
Number of falls	Formula display time	-0.237958	0.00167	0.008884	0.015433	0.909285	0.986671
Number of falls	Display time increments	0.204406	0.007152	0.034197	0.127453	0.344764	0.659361
Number of falls	Display time decrements	-0.251432	0.000878	0.005838	0.112885	0.40312	0.694069

Appendix 1-Table 7 (continued): Correlations for Figure 3b.

Variable name	Variable name	rm-correlation	p-value	p-value BH corrected	Spearman correlation	p-value	p-value BH corrected
Distance travelled	ΔHeart rate	-0.019502	0.799553	1	0.167682	0.21248	0.543197
Distance travelled	ΔHRV (lf/hf)	0.043124	0.574321	0.966493	-0.167488	0.213018	0.543197
Distance travelled	ΔHeart rate deceleration capacity	-0.139427	0.068128	0.24241	-0.019769	0.883951	0.972982
Distance travelled	ΔHRV (triangular index)	0.07714	0.314508	0.711306	0.030529	0.821641	0.946911
Distance travelled	ΔRespiration rate	0.277974	0.000222	0.001891	0.196461	0.143002	0.50882
Distance travelled	ΔSCRcda	0.14533	0.057146	0.21325	0.169886	0.206445	0.543197
Distance travelled	Performance	0.095892	0.210815	0.520236	0.169043	0.208738	0.543197
Distance travelled	Competence	-0.261945	0.000518	0.003965	0.214091	0.109788	0.409697
Distance travelled	Response latency	0.311406	0.000032	0.000327	-0.191405	0.153785	0.534753
Distance travelled	Formula display time	0.309827	0.000035	0.000338	-0.242481	0.069162	0.316445
Distance travelled	Display time increments	-0.023966	0.754988	1	-0.274371	0.038887	0.228833
Distance travelled	Display time decrements	0.131314	0.085969	0.292293	-0.170145	0.205743	0.543197
Distance travelled	Number of falls	0.03144	0.682226	1	-0.28514	0.031561	0.201205
Number of tiles visited	ΔHeart rate	0.106197	0.165584	0.452398	0.149296	0.267683	0.603393
Number of tiles visited	ΔHRV (lf/hf)	0.0487	0.525813	0.917881	-0.186158	0.165602	0.535886
Number of tiles visited	ΔHeart rate deceleration capacity	-0.034079	0.65718	1	0.028717	0.832069	0.950049
Number of tiles visited	ΔHRV (triangular index)	0.115078	0.13278	0.395774	0.004218	0.975156	0.990496
Number of tiles visited	ΔRespiration rate	0.195906	0.010008	0.045036	0.153741	0.253533	0.603393
Number of tiles visited	ΔSCRcda	0.124481	0.103742	0.337712	0.180414	0.179282	0.535886
Number of tiles visited	Performance	0.019262	0.801972	1	0.180836	0.17825	0.535886
Number of tiles visited	Competence	-0.241243	0.001432	0.008117	0.20381	0.128348	0.467553
Number of tiles visited	Response latency	0.257916	0.000636	0.004422	-0.169382	0.207815	0.543197
Number of tiles visited	Formula display time	0.242194	0.00137	0.008117	-0.220748	0.09892	0.388071
Number of tiles visited	Display time increments	0.037547	0.624834	1	-0.254624	0.055951	0.286123
Number of tiles visited	Display time decrements	0.086104	0.261399	0.633975	-0.116523	0.388036	0.694069
Number of tiles visited	Number of falls	0.17434	0.022177	0.094254	-0.332297	0.011556	0.097965

Appendix 1-Table 7 (continued): Correlations for Figure 3b.

Variable name	Variable name	rm-correlation	p-value	p-value BH corrected	Spearman correlation	p-value	p-value BH corrected
Number of tiles visited	Distance travelled	0.595722	0	0	0.932605	0	0
Time in center	ΔHeart rate	0	1	1	-0.128942	0.33911	0.656758
Time in center	ΔHRV (lf/hf)	0	1	1	-0.042041	0.756176	0.906548
Time in center	ΔHeart rate deceleration capacity	0	1	1	0.123367	0.360577	0.672784
Time in center	ΔHRV (triangular index)	0	1	1	0.093903	0.487191	0.694069
Time in center	ΔRespiration rate	0	1	1	0.050144	0.711065	0.870343
Time in center	ΔSCRcda	0	1	1	0.074228	0.583177	0.747846
Time in center	Performance	0	1	1	0.092347	0.494467	0.694069
Time in center	Competence	0	1	1	0.223526	0.094639	0.381047
Time in center	Response latency	0	1	1	-0.265729	0.045734	0.249904
Time in center	Formula display time	0	1	1	-0.298175	0.024271	0.161454
Time in center	Display time increments	0	1	1	-0.177855	0.185634	0.535886
Time in center	Display time decrements	0	1	1	-0.129331	0.337643	0.656758
Time in center	Number of falls	0	1	1	0.024567	0.856059	0.956037
Time in center	Distance travelled	0	1	1	0.072931	0.589791	0.747846
Time in center	Number of tiles visited	0	1	1	-0.00826	0.951377	0.990496
Time in edges	ΔHeart rate	-0.073491	0.338015	0.717837	0.128942	0.33911	0.656758
Time in edges	ΔHRV (lf/hf)	-0.032298	0.674039	1	0.042041	0.756176	0.906548
Time in edges	ΔHeart rate deceleration capacity	-0.001157	0.987982	1	-0.123367	0.360577	0.672784
Time in edges	ΔHRV (triangular index)	-0.023398	0.760622	1	-0.093903	0.487191	0.694069
Time in edges	ΔRespiration rate	0.050678	0.509118	0.905757	-0.050144	0.711065	0.870343
Time in edges	ΔSCRcda	0.11382	0.137098	0.395774	-0.074228	0.583177	0.747846
Time in edges	Performance	0.075838	0.322769	0.715705	-0.092347	0.494467	0.694069
Time in edges	Competence	-0.024293	0.751757	1	-0.223526	0.094639	0.381047
Time in edges	Response latency	0.110861	0.147674	0.410803	0.265729	0.045734	0.249904

Appendix 1-Table 7 (continued): Correlations for Figure 3b.

Variable name	Variable name	rm-correlation	p-value	p-value BH corrected	Spearman correlation	p-value	p-value BH corrected
Time in edges	Formula display time	0.084987	0.267656	0.633975	0.298175	0.024271	0.161454
Time in edges	Display time increments	-0.116196	0.129032	0.394837	0.177855	0.185634	0.535886
Time in edges	Display time decrements	0.00295	0.969364	1	0.129331	0.337643	0.656758
Time in edges	Number of falls	-0.06455	0.400194	0.77506	-0.024567	0.856059	0.956037
Time in edges	Distance travelled	-0.008131	0.915689	1	-0.072931	0.589791	0.747846
Time in edges	Number of tiles visited	0.160263	0.035721	0.143823	0.00826	0.951377	0.990496
Time in edges	Time in center	-1	0	0	-1	0	0
Time in corners	ΔHeart rate	0	1	1	0.012052	0.929098	0.990496
Time in corners	ΔHRV (lf/hf)	0	1	1	-0.241507	0.070321	0.316445
Time in corners	ΔHeart rate deceleration capacity	0	1	1	0.103465	0.443736	0.694069
Time in corners	ΔHRV (triangular index)	0	1	1	0.012019	0.929291	0.990496
Time in corners	ΔRespiration rate	0	1	1	-0.07261	0.591434	0.747846
Time in corners	ΔSCRcda	0	1	1	0.038824	0.774319	0.918378
Time in corners	Performance	0	1	1	0.077253	0.567876	0.747846
Time in corners	Competence	0	1	1	0.001614	0.990496	0.990496
Time in corners	Response latency	0	1	1	-0.006553	0.961414	0.990496
Time in corners	Formula display time	0	1	1	-0.001877	0.988944	0.990496
Time in corners	Display time increments	0	1	1	0.088878	0.510893	0.704204
Time in corners	Display time decrements	0	1	1	-0.023347	0.863134	0.956953
Time in corners	Number of falls	0	1	1	-0.184961	0.168387	0.535886
Time in corners	Distance travelled	0	1	1	0.232945	0.081192	0.345064
Time in corners	Number of tiles visited	0	1	1	0.319434	0.015432	0.118057
Time in corners	Time in center	0	1	1	-0.502393	0.000068	0.000871
Time in corners	Time in edges	0	1	1	0.502393	0.000068	0.000871

Appendix 1-Table 8: Statistical tests for Appendix 1-figure S3c-h. Repeated measures ANOVA (4 by 4), within- and between-subjects' effects, for the Group (quartiles) x Block (T1, T2, T3, T4) model design, to predict each one of the dependent variables in Figures 2e-j. Greenhouse-Geisser sphericity correction applied to all tests due to sphericity violations, as seen from the Mauchly's test p-values.

**Within-subjects effects**								
**Dependent variable**	**Mauchly's test p-value**	**Independent variables**	**Sum of squares**	**df**	**Mean square**	**F-statistic**	**p-value**	**η^2_p**
Respiration rate (cpm)	1.5705e-5	Block	25.5	2.16	11.79	3.380	0.033993	0.060
		Block*Group	12.4	6.49	1.91	0.549	0.783122	0.030
		Residual	399.8	114.59	3.49			
SCR (rpm)	0.16307	Block	179	3	59.8	1.693	0.17063	0.031
		Block*Group	240	9	26.7	0.757	0.65637	0.041
		Residual	5614	159	35.3			
Heart rate (bpm)	9.8913e-13	Block	183	1.85	98.8	3.829	0.028049	0.067
		Block*Group	132	5.55	23.7	0.920	0.478379	0.050
		Residual	2529	98.00	25.8			
Heart deceleration capacity (bpm)	0.11052	Block	44.1	3	14.69	2.792	0.042287	0.050
		Block*Group	40.8	9	4.53	0.862	0.560959	0.046
		Residual	836.6	159	5.26			
HRV (lf/hf)	0.051616	Block	9.42	3	3.140	1.427	0.23696	0.026
		Block*Group	7.50	9	0.833	0.378	0.94408	0.021
		Residual	349.87	159	2.200			
HRV (triangular index)	0.019053	Block	87.1	2.56	34.06	8.527	8.6841e-5	0.139
		Block*Group	27.5	7.67	3.59	0.898	0.51690	0.048
		Residual	541.0	135.45	3.99			

**Between-subjects effects**							
**Dependent variable**	**Independent variables**	**Sum of squares**	**df**	**Mean square**	**F-statistic**	**p-value**	**η^2_p**
Respiration rate (cpm)	Group	2.32	3	0.775	0.0172	0.99689	0.001
	Residual	2387.80	53	45.053			
SCR (rpm)	Group	271	3	90.2	0.511	0.67624	0.028
	Residual	9350	53	176.4			
Heart rate (bpm)	Group	984	3	328	0.564	0.64106	0.031
	Residual	30818	53	581			
Heart deceleration capacity (bpm)	Group	83.9	3	28.0	0.379	0.76883	0.021
	Residual	3913.7	53	73.8			
HRV (lf/hf)	Group	34.3	3	11.44	1.23	0.30828	0.065
	Residual	493.5	53	9.31			
HRV (triangular index)	Group	67.3	3	22.4	0.647	0.58844	0.035
	Residual	1837.1	53	34.7			

Note. Type 3 Sums of Squares

Appendix 1-Table 9: Spearman correlations between behavioral variables and cortisol AUCi.

	Spearman correlation	p-value	p-value BH corrected
Performance	-0.171247	0.202778	0.486668
Competence	-0.215647	0.107170	0.412303
Response latency	0.199183	0.137434	0.412303
Formula display time	0.233212	0.080834	0.412303
Display time increments	0.076744	0.570441	0.740366
Display time decrements	-0.067669	0.616972	0.740366
Number of falls	-0.300407	0.023177	0.278129
Distance travelled	-0.007324	0.956877	0.956877
Number of tiles visited	-0.008956	0.947284	0.956877
Time in center	-0.143204	0.287915	0.493569
Time in edges	0.143204	0.287915	0.493569
Time in corners	0.095990	0.477519	0.716279

Appendix 1-Table 10: Comparison between IMVEST and other popular stress protocols in terms of stress markers (cortisol, HR, HRV and EDA) both as increase and differences with a control group. In order to address this question, we have analyzed effect sizes from IMVEST, TSST, VR-TSST, SECPT, MIST, MAST studies including reported statistics. Note that we could only identify a few valid studies for this pilot meta-analysis, as many reports do not include enough information (e.g., effect size, etc). As can be seen, IMVEST effects on heart rate and electrodermal activity are either in line or larger than those reported in other studies, while the cortisol response slightly lower as expected from the timing of saliva sampling.

Variable	Comparison	IMVEST	TSST	VR-TSST	SPECT	MAST	MIST
Cortisol	Increase	d=0.27	*1: d=0.87 *2: d=0.67				*7: d=0.64
	Control contrast	d=0.48	*2: d=0.79	*3: d=0.74	*5: d=1.22	*6: d=1.50	*7: d=3.12
HR	Increase	d=1.51	*2: D=1.20	*3: d=2.35	*5: n.s.		
	Control contrast	d=0.79	*2: D=0.73	*3: n.s.	*5: n.s.		
HRV	Increase	d=1.51					
	Control contrast	d=0.82					
EDA	Increase	d=0.41		*3: d=0.74 *4: d=0.41			
	Control contrast	d=0.40		*3: n.s.			

*1 Average extracted from (Dickerson and Kemeny, 2004).
*2 Effect size computed from means and standard deviations in the supplemental materials of (Woody et al., 2018).
*3 Effect size computed from reported effect sizes in ANOVA tests in (Zimmer et al., 2019). For both HR and EDA, increases occurred for both control and stress conditions so the “Increase” effect size reported are not due to the stress condition [Control contrast effects are non-significant (n.s.)]
*4 Effect size computed from mean and standard deviation reported in (Montero-López et al., 2015)
*5 Effect size computed from reported effect sizes in ANOVA tests in (Schwabe et al., 2008). Effects on HR are not significant (n.s.) however, there are strong effects in blood pressure, not included here for compatibility reasons.
*6 Effect size computed from the reported effect sizes in ANOVA tests in Study 3 from (Smeets et al., 2012).
*7 Effect size computed from the reported ANOVA’s F scores and sample sizes from Study 1 and Study 3 in (Dedovic et al., 2005).

Supplementary Figures

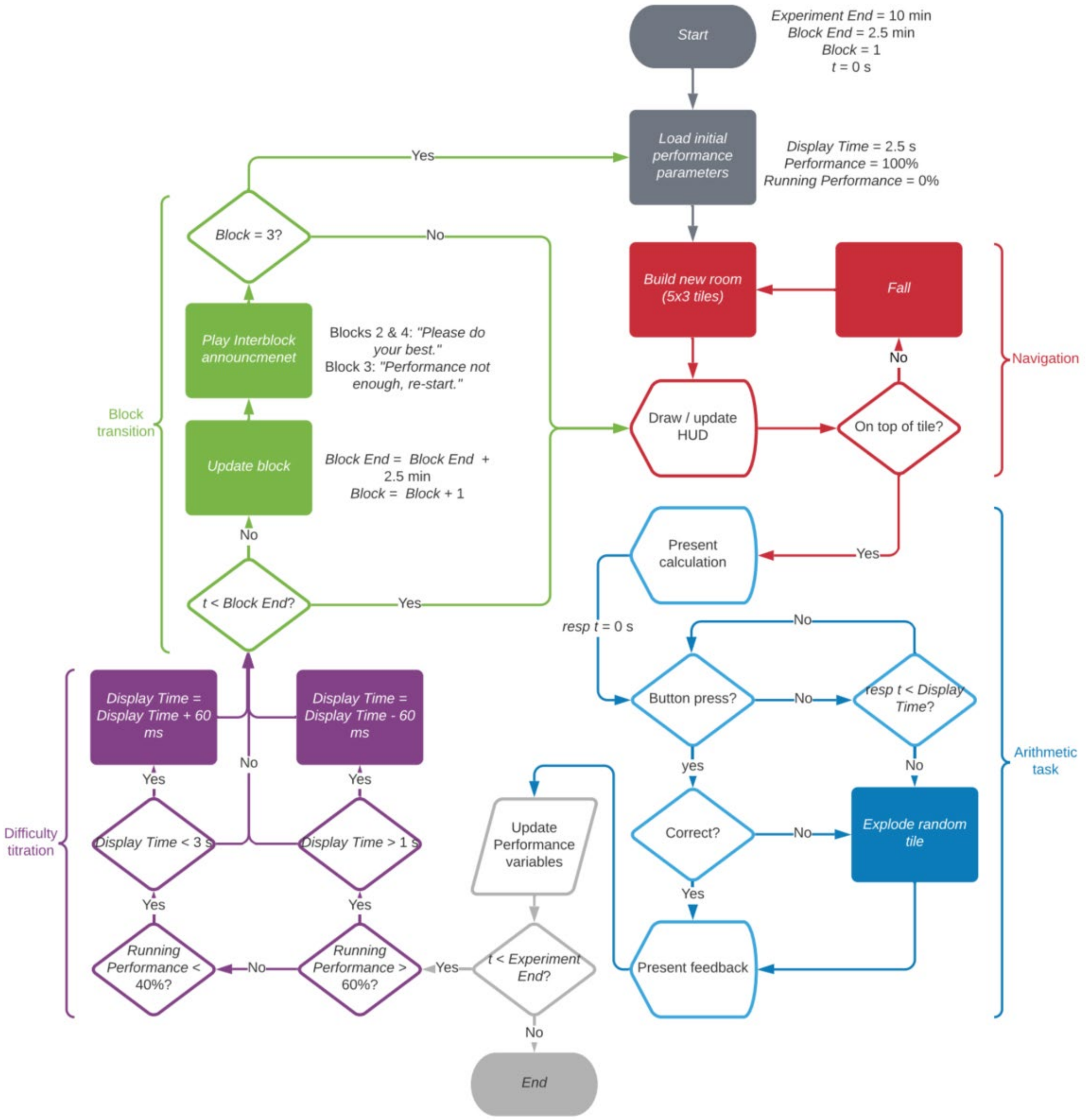

Figure S1: Flowchart for the program logic governing the stress test, depicted in Figure 1a. The logic encompasses navigation elements, arithmetic task and evaluation, difficulty titration and block transitions.

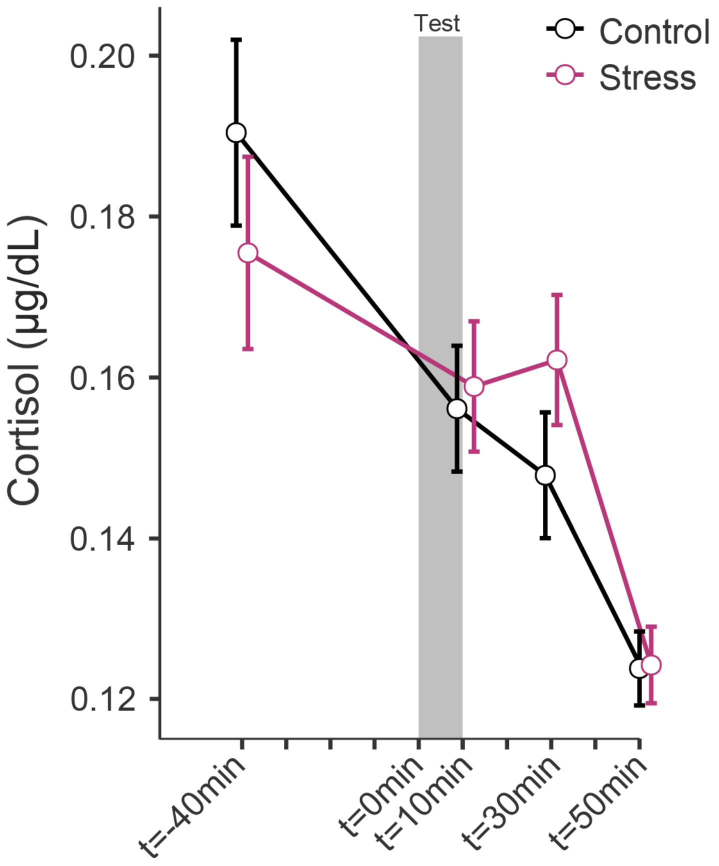

Figure S2: Raw cortisol values between stress and control groups.

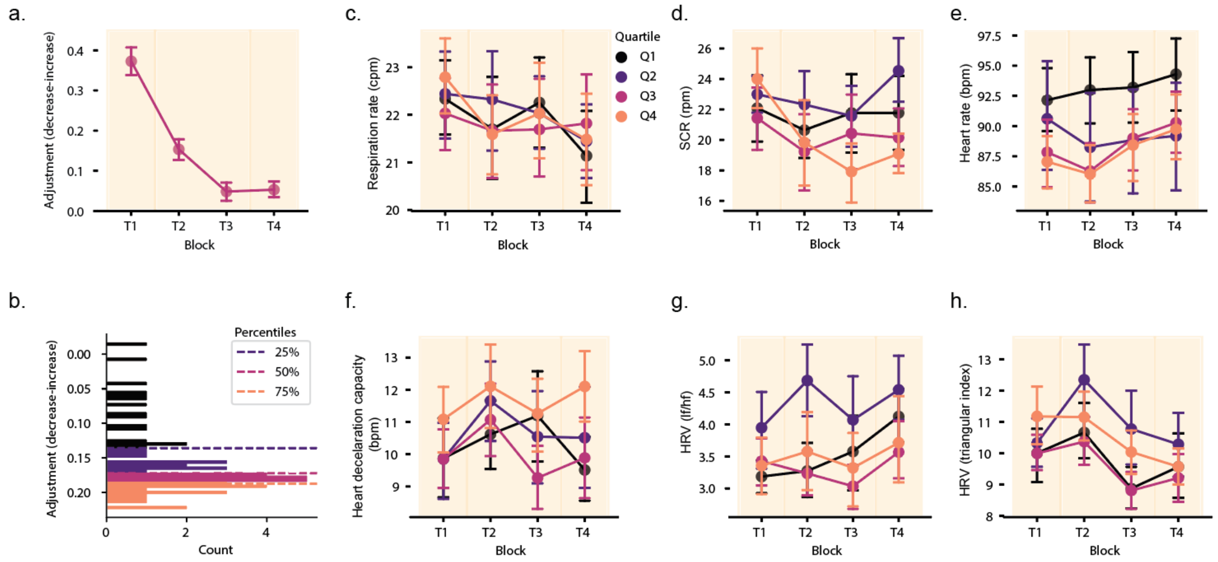

Figure S3: Balance in difficulty adjustments in IMVEST and its relation with physiological variables. (a) Adjustment balance, defined as the difference between decrements and increments in the formula display time, per individual and per block. A higher value indicates a larger number of display time decrements than increments. Prevalence of decrements is larger in the initial blocks. (b) Separation of IMVEST participants into quartiles based on their average value of adjustments. (c-h) Physiological activations between the quartile groups.

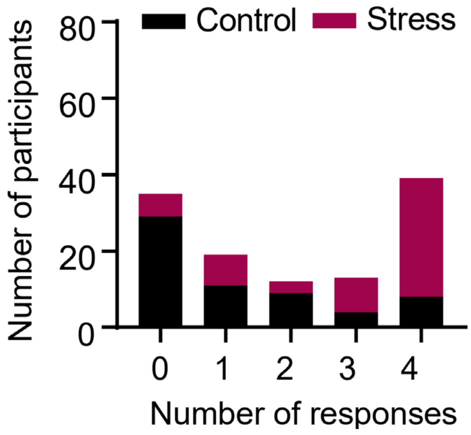

Figure S4: Proportion of participants being classified as responders with different number of responses in the control and stress groups. Following the procedure described in Materials and Methods, we defined the number of physiological responses for each individual, as the number of the T1-T4 blocks classified as belonging to a stress condition (PLSR model score above the individual threshold). We identified 6 non-responders (0 blocks classified as stress) out of the 57 participants in the stress group and 29 out of the 61 participants in the control group. As expected, more blocks are classified as *stress* in the experimental group [$X^2(4, N=118) = 34.0, p<0.001$].

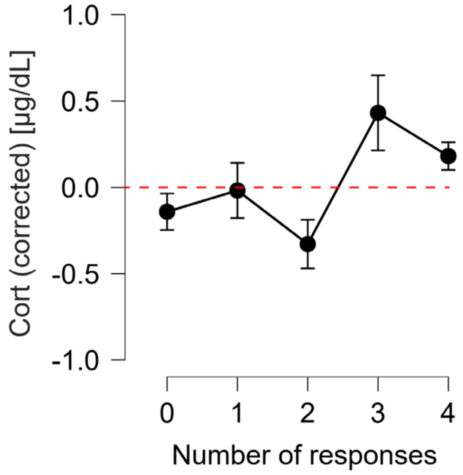

Figure S5: Cortisol response for participants with different number of physiological responses. Number of responses has an effect on cortisol (one-way ANOVA; $F_{1,116}$=3.81, p =0.006, η_p^2 = 0.120). A linear contrast analysis suggests that participants with higher number of physiological responses also have larger cortisol responses (linear contrast *t*=3.1, p=0.003).

Appendix 1-Algorithm S1: Identification of the number of physiological responses. Participants is the set with all {1,...,*N*x*M*} *N* participants and *M* experimental blocks, for all physiology variables and corresponding experimental group. Participant_n is the set with the *M* experimental blocks for participant *n*.

```
FOR each Participant_n in Participants
    SET Training as Participants EXCEPT Participant_n
    SET TrainingReduced as FeatureSelection on Training (if feature selection is used)
    FOR each Participant_j in TrainingReduced
        SET TrainingLOO as TrainingReduced EXCEPT Participant_j
        FIT PLSR model to TrainingLOO as PLSR_j
        PREDICT Participant_j with PLSR_j as predictions_j
        STORE predictions_j in PredicitonsLOO
    END FOR
    FIT ROC curve to PredicitonsLOO
    GET falsePositives, truePositives and thresholds from ROC curve
    SET J as truePositives – falsePositives
    SET idx as index of MAX value in J
    SET optimalThreshold_n as thresholds(idx)
    FIT PLSR to Training as PLSR_n
    PREDICT Participant_n with PLSR_n as predictions_n
    SET numResponses_n as COUNT(predictions_n < optimalThreshold_n)
END FOR
```

References

Dedovic, K., Renwick, R., Mahani, N.K., Engert, V., Lupien, S.J., Pruessner, J.C., 2005. The Montreal Imaging Stress Task: Using functional imaging to investigate the effects of perceiving and processing psychosocial stress in the human brain. J. Psychiatry Neurosci. 30, 319–325.

Dickerson, S.S., Kemeny, M.E., 2004. Acute stressors and cortisol responses: A theoretical integration and synthesis of laboratory research. Psychol. Bull. 130, 355–391. https://doi.org/10.1037/0033-2909.130.3.355

Montero-López, E., Santos-Ruiz, A., García-Ríos, M.C., Rodríguez-Blázquez, R., Pérez-García, M., Peralta-Ramírez, M.I., 2015. A virtual reality approach to the Trier Social Stress Test: Contrasting two distinct protocols. Behav. Res. Methods 223–232. https://doi.org/10.3758/s13428-015-0565-4

Schwabe, L., Haddad, L., Schachinger, H., 2008. HPA axis activation by a socially evaluated cold-pressor test. Psychoneuroendocrinology 33, 890–895. https://doi.org/10.1016/j.psyneuen.2008.03.001

Smeets, T., Cornelisse, S., Quaedflieg, C.W.E.M., Meyer, T., Jelicic, M., Merckelbach, H., 2012. Introducing the Maastricht Acute Stress Test (MAST): A quick and non-invasive approach to elicit robust autonomic and glucocorticoid stress responses. Psychoneuroendocrinology 37, 1998–2008. https://doi.org/10.1016/j.psyneuen.2012.04.012

Woody, A., Hooker, E.D., Zoccola, P.M., Dickerson, S.S., 2018. Social-evaluative threat, cognitive load, and the cortisol and cardiovascular stress response. Psychoneuroendocrinology 97, 149–155. https://doi.org/10.1016/j.psyneuen.2018.07.009

Zimmer, P., Buttlar, B., Halbeisen, G., Walther, E., Domes, G., 2019. Virtually stressed? A refined virtual reality adaptation of the Trier Social Stress Test (TSST) induces robust endocrine responses. Psychoneuroendocrinology 101, 186–192. https://doi.org/10.1016/j.psyneuen.2018.11.010
